# YOLO-Based Vision Perception and Automation for Agricultural Robots: A Comprehensive Survey of Algorithms, Applications, and Deployment

**DOI:** 10.3390/s26206637

**Published:** 2026-10-20

**Authors:** Hanzhi Ping, Zichen Zhang, Caixia Song, Tao Ping

**Affiliations:** 1 College of Intelligent Equipment, Shandong University of Science and Technology, Qingdao 266100, China; 2 College of Science and Information, Qingdao Agricultural University, Qingdao 266109, China; 3 Department of Science and Technology, Qingdao Agricultural University, Qingdao 266109, China

**Keywords:** YOLO, agricultural robotics, deep learning, object detection, computer vision, precision agriculture, edge computing, autonomous navigation

## Abstract

Deep learning-driven computer vision has become a fundamental enabling technology for agricultural robotics, providing reliable perception capabilities for precision farming and autonomous field operations. Among various object detection frameworks, the You Only Look Once (YOLO) family has attracted extensive attention due to its favorable balance between detection accuracy, computational efficiency, and real-time deployment capability in complex and dynamic agricultural environments. This paper presents a comprehensive review of YOLO-based vision perception and automation technologies for agricultural robotic systems. The evolution of the YOLO framework from YOLOv1 to recent YOLOv11 variants is systematically reviewed, with particular emphasis on architectural innovations, performance improvements, and adaptations to agriculture-specific challenges, including complex backgrounds, illumination variations, severe occlusion, and multi-scale small object detection. Beyond algorithm-level analysis, this survey investigates the integration of YOLO-based perception modules into robotic systems for practical applications, including autonomous navigation, precision spraying, crop monitoring, disease detection, and robotic harvesting. Furthermore, this review analyzes the complete deployment pipeline of YOLO-based agricultural robots, covering benchmark datasets, evaluation metrics, model optimization strategies, and hardware constraints for edge computing platforms. Emerging research directions are also discussed, including lightweight YOLO architectures, multimodal vision–language models, and cloud–edge collaborative intelligence for next-generation agricultural automation. By bridging advances in computer vision algorithms with robotic perception and execution, this survey provides a comprehensive perspective on the development of intelligent and efficient agricultural robotic systems.

## 1. Introduction

The confluence of exponential population growth and diminishing arable land poses a formidable global challenge: maximizing agricultural productivity to ensure food security while simultaneously minimizing resource wastage and ecological footprints [1,2]. To mitigate these pressing constraints, the paradigm shift toward precision agriculture and intelligent robotic systems has escalated from an experimental pursuit to an operational imperative. Within this domain, computer vision technology serves as the sensory cornerstone, enabling autonomous platforms to execute sophisticated, high-precision tasks under field conditions. In particular, the You Only Look Once (YOLO) algorithm family has emerged as the vanguard framework, empowering robust robotic perception and real-time automation within dynamic, highly nonlinear, and unstructured agricultural environments [3].

Since its seminal inception by Redmon et al. in 2015 [4], the YOLO framework has undergone a rigorous evolutionary trajectory spanning from the foundational YOLOv1 to the state-of-the-art YOLOv11. This continuous architectural maturation has introduced pioneering innovations in deep backbone design, multi-scale feature fusion necks, NMS-free training strategy, and attention-driven optimization techniques, substantially bolstering both detection fidelity and computational efficiency [5].

In the arena of smart farming and agricultural robotics, this evolutionary lineage has sequentially unlocked solutions to domain-specific bottlenecks. Early iterations, such as YOLOv3, demonstrated notable utility in real-time weed detection systems by processing aerial video streams from unmanned aerial vehicles (UAVs) to guide localized herbicide application [6,7]. Subsequently, YOLOv4 enhanced model resilience against complex, heavily textured agricultural backdrops. By incorporating advanced topological components—such as the Cross Stage Partial Darknet53 (CSPDarknet53) backbone, Spatial Pyramid Pooling (SPP), Path Aggregation Network (PAN), and Cross-Iteration Batch Normalization (CBN)—coupled with the Spatial Attention Module (SAM), YOLOv4 successfully streamlined the training pipeline while keeping the high-speed execution viable for robotic integration [8].

The transition to the PyTorch-based YOLOv5 marked a pivotal milestone toward modularity, structural flexibility, and rapid deployment. Featuring a Cross Stage Partial (CSP) Net backbone and multiple SPP blocks, YOLOv5 facilitated robust multi-scale feature extraction and map resolution upsampling [9,10]. Thanks to its favorable balance between accuracy and inference speed, YOLOv5 provided the real-time inference latency critical for the autonomous navigation and field operations of ground-based robotic agents.

To conquer the unique geometric and environmental constraints inherent to modern agronomic settings—such as severe canopy foliage occlusion, varying illumination, and multi-scale targets—the latter generations introduced profound algorithmic modifications. YOLOv8 addressed the structural irregularities and morphology variations of agricultural targets (e.g., dense grape clusters or diseased leaf lesions) through an anchor-free detection paradigm, thereby circumventing the adaptability limitations typical of traditional anchor-based precursors [11,12]. To further compress inference latency, YOLOv10 eliminated the computational bottleneck associated with Non-Maximum Suppression (NMS) via a consistent dual-label assignment strategy for NMS-free training, capturing salient feature maps while drastically reducing processing overhead [13,14]. This latency reduction is highly advantageous for agile tasks like orchard robotic harvesting. Most recently, YOLOv11 has redefined the Pareto frontier of the speed–accuracy trade-off by embedding innovative structural primitives, including the Cross Stage Partial Network with 2 Kernels (C3k2), Spatial Pyramid Pooling-Fast (SPPF), and Cross-Level Pyramid Slice Attention (C2PSA) [15]. These hardware-conscious architectural refinements enable scalable deployment from low-power edge-computing microcontrollers to distributed cloud-computing nodes, facilitating the realization of a seamless perception-to-execution loop in real-time robotic automation.

Concurrently, the implementation spectrum of the YOLO ecosystem within precision agriculture has expanded from macro-level field monitoring to micro-level phenotypic actuation. Specifically, YOLO-driven perception engines have witnessed widespread adoption across several critical sub-domains:

**Weed Localization and Precision Spraying**: Weed Localization and Precision Spraying: Enabling high-fidelity differentiation between specific crop varieties and invasive weed species, providing a technical foundation for potential reductions in pesticide use [6].

**Phytopathology and Disease Detection**: Identifying early-stage crop disease manifestations and localized leaf lesions to prevent widespread orchard contamination [16].

**Yield Estimation and Crop Phenotyping**: Analyzing target growth stages, spatial distributions, and fruit counts to deliver actionable data-driven insights for yield forecasting [17].

**Livestock Management and Behavioral Analysis**: Monitoring the posture, spatial density, and physiological health status of livestock in intensive farming facilities [5,18].

Despite the ubiquitous proliferation of YOLO-based models in agricultural research, a critical literature gap remains. Prior surveys predominantly focus on fragmented, case-specific vision algorithms evaluated under standard machine learning metrics (e.g., mAP), leaving a conceptual and engineering disconnection between isolated object detection and downstream robotic control loops. Terven et al. [3] provided a technical review of YOLO architectures from v1 to v8 but did not address agricultural robot application scenarios. Alif and Hussain [5] surveyed YOLO applications in agriculture but focused on crop yield prediction and disease identification, without covering the robotic execution layer or edge deployment. Sapkota et al. [19] conducted a decade review of the YOLO series, primarily focusing on algorithm evolution, lacking systematic analysis of agriculture-specific challenges. Zohaib et al. [20] reviewed object detection methods in agriculture but did not delve into YOLO integration with large models or the robotic closed-loop system. Furthermore, a systematic taxonomy that maps the historical evolution of the entire YOLO family against specific agricultural constraints (e.g., severe illumination variations, occlusion, and edge-hardware limitations) is currently lacking.

To bridge this critical academic and technical chasm, this paper provides a comprehensive, state-of-the-art survey of the YOLO ecosystem tailored specifically for agricultural robotics. The primary contributions of this review are three-fold:

(1) **Comprehensive Algorithmic Taxonomy**: We present a rigorous chronological and architectural analysis of the YOLO lineage (v1 through v11), systematically decoding the domain-specific network optimizations designed to combat extreme field constraints.

(2) **Perception-to-Execution Actuation Loop**: We delineate the methodology for seamlessly embedding YOLO frameworks into real-time robotic control pipelines, evaluating their impact on closed-loop autonomous navigation, precision spraying, and robotic harvesting.

(3) **Edge Ecosystem and Future Roadmap**: We meticulously evaluate the deployment challenges on resource-constrained embedded edge hardware, and formulate a forward-looking roadmap highlighting the synergy of lightweight YOLO models with multimodal large language models and cloud–edge collaborative architectures.

The remainder of this survey is organized as follows. Section 2 outlines the literature methodology and research design. Section 3 reviews the core architecture of agricultural robot systems. Section 4 traces the chronological evolution of the YOLO family and analyzes algorithmic optimizations for field constraints. Section 5 explores the emergence and applications of agricultural large vision–language models. Section 6 evaluates the integration of YOLO perception into downstream robotic actuation loops, focusing on navigation, spraying, and harvesting. Section 7 addresses the implementation ecosystem, including datasets, metrics, and edge hardware deployment. Section 8 discusses engineering bottlenecks and outlines a roadmap for intelligent agriculture. Finally, Section 9 summarizes future trends and concludes the paper. For clarity, the main abbreviations and symbols used throughout this survey are summarized in Table 1.

## 2. Content Analysis Method and Research Process Design

### 2.1. Sample Extraction

Generally, journal articles can more swiftly and directly capture research trends and cutting-edge developments when compared to books, research reports, and academic papers [21]. Thus, the guidelines for sampling are as follows:

**Paper Collection:** We primarily utilize Google Scholar and Baidu Scholar as our main search engines. Additionally, we have employed the databases China National Knowledge Infrastructure (CNKI) (https://www.cnki.net/), Web of Science, and Institute of Electrical and Electronics Engineers (IEEE) Xplore as three crucial tools for discovering relevant papers.

**Time Interval:** From 2016 to 2026.

**Major Search Keywords:** The major search keywords are “YOLO Algorithm”, “YOLO”, “Agriculture Robots”, “large model” Crop Monitoring”, “Pest and Disease Detection”, “Yield Estimation”, “Resource Optimization”, “Livestock Management”, “Weed Detection”, etc.

**Content Analysis Coding:** According to the research objectives of this paper, the six researchers discuss and set the analysis coding rule together.

**Basic Information of the Papers:** Title, author, year of publication, the journal name, technique used, applied model, specific content of the model study.

**Research Content Analysis:** Analysis of the number of YOLO algorithms, analysis of advantages and disadvantages of YOLO algorithm, and analysis of YOLO algorithm technology application.

**Boolean Search Strings:** The search strings used in Google Scholar and Web of Science are: (“YOLO” OR “You Only Look Once”) AND (“agricultural robot*” OR “farming robot*” OR “precision agriculture” OR “smart farming”) AND (“object detection” OR “weed detection” OR “disease detection” OR “yield prediction” OR “livestock monitoring”).

**Inclusion and Exclusion Criteria:** We defined explicit inclusion and exclusion criteria before screening. Studies were included only if they met all of the inclusion criteria, while any study matching one or more exclusion criteria was removed. The detailed criteria are summarized in Table 2.

### 2.2. Research Steps

**Figure 1:**
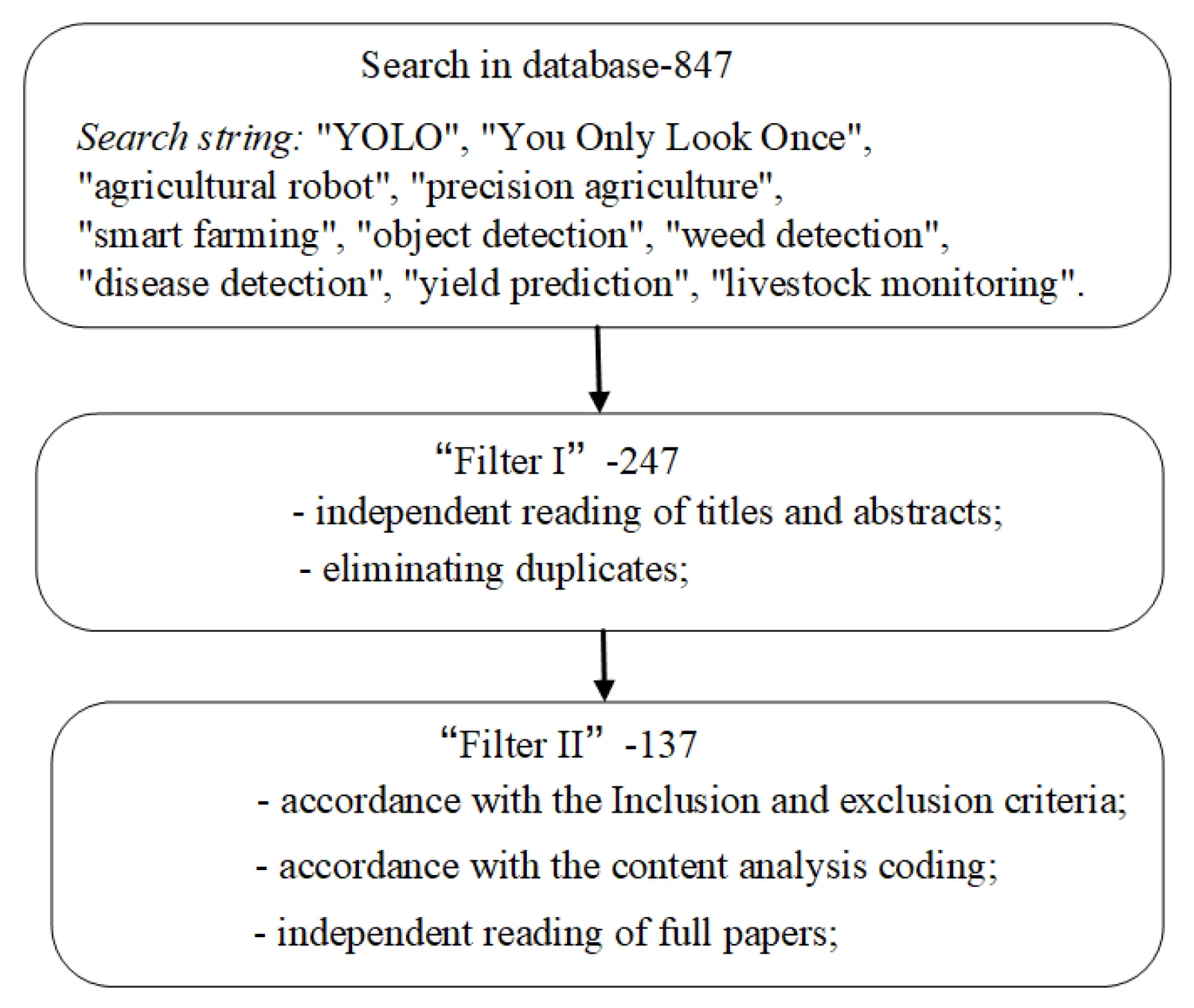
Methodology of the literature screening and sample extraction process.

**Research Steps Overview:** The overall literature screening and sample extraction process is illustrated in Figure 1. It consists of seven steps, starting from the initial database search and proceeding through deduplication, title and abstract screening, full-text screening, backward citation searching, and final classification. The detailed steps are described below.

**Step One:** According to the principle of sample extraction, papers were extracted and screened, and then 847 initial samples were obtained.

**Step Two:** Literature search was conducted. Zotero was used for automatic deduplication by DOI and title, followed by manual verification. Screening disagreements were arbitrated before full-text review, data extraction and quality assessment. Papers adopting YOLO algorithm were included in statistical samples; others were excluded.

**Step Three:** The technologies used in the papers and their application models were identified. This step was done independently by four authors.

**Step Four:** The preliminary identification results of the two researchers were combined. And the identification results that were controversial were discussed and determined by four authors.

**Step Five:** The preliminary classification was performed by four authors.

**Step Six:** The controversial classifications were discussed and determined by four authors, and finally the final research samples were obtained.

### 2.3. Quantitative Analysis

**Figure 2:**
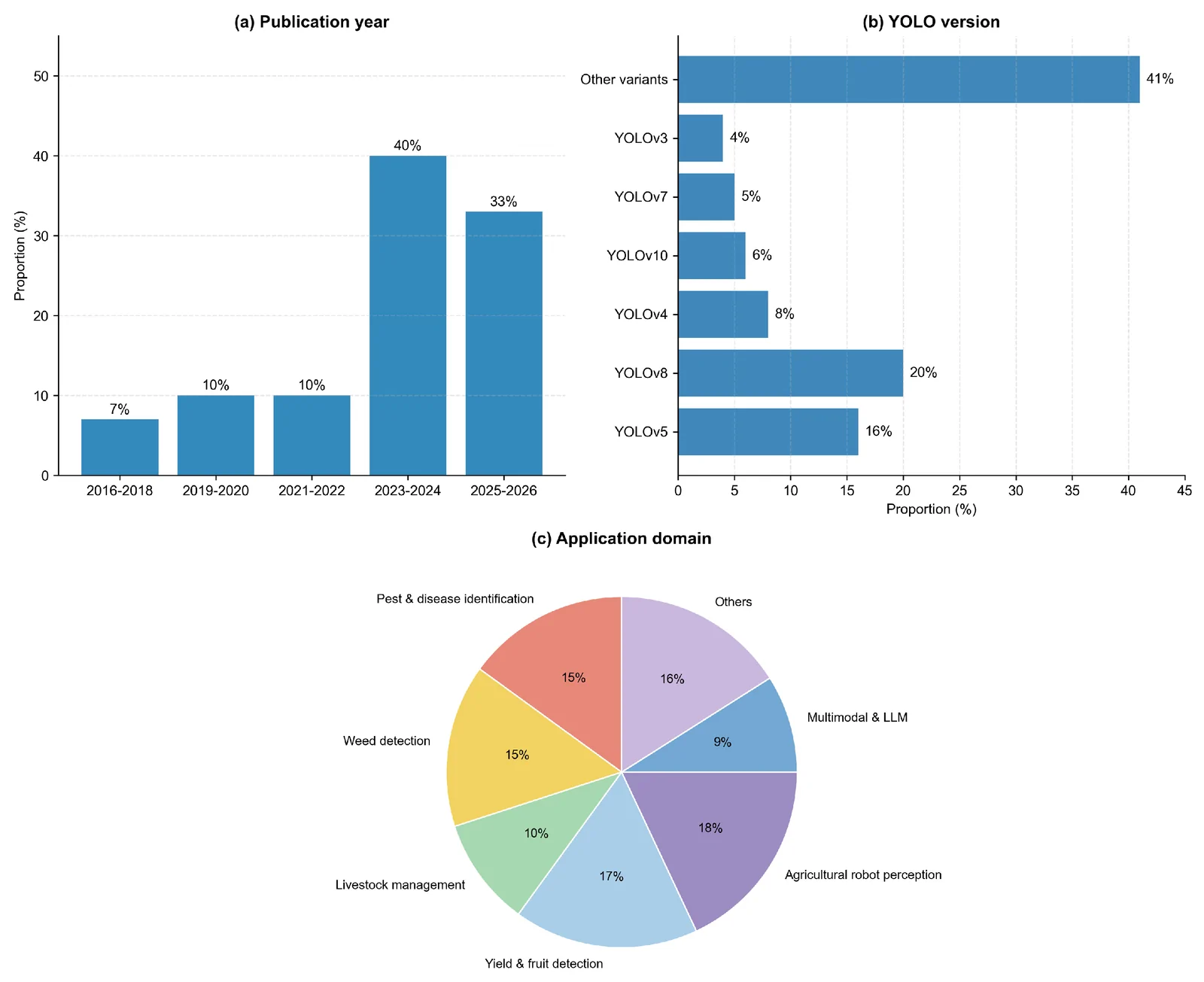
Quantitative distribution of the 137 included papers.

This section provides a quantitative statistical analysis of the 137 agricultural YOLO studies included in this paper. Figure 2 summarizes their distribution by YOLO version, application domain, and publication year. YOLOv8-related studies account for approximately 20%, followed by YOLOv5 (16%), YOLOv4 (8%), YOLOv10 (6%), YOLOv7 (5%), YOLOv3 (4%), and other YOLO variants (41%). By application domain, pest and disease identification accounts for 15%, weed detection 15%, livestock management 10%, yield and fruit target detection 17%, agricultural robot perception 18%, multimodal and large-model research 9%, and others 16%.

By publication year, the studies are distributed as 2016–2018 (7%), 2019–2020 (10%), 2021–2022 (10%), 2023–2024 (40%), and 2025–2026 (33%), showing rapid growth particularly after the release of YOLOv5, YOLOv8 and subsequent YOLO series. The research trend reveals a clear shift from simple object detection tasks toward complex, practical agricultural scenarios. Early studies primarily focused on weed detection and disease identification with relatively simple backgrounds; after 2020, livestock management, fruit detection and harvesting robotics emerged, reflecting growing demands for on-site agricultural automation. After 2023, studies on newer YOLO versions (YOLOv9-v12), YOLO-large vision–language model integration began to increase, indicating that researchers are gradually exploring the combination of lightweight detection models and multimodal large models to tackle challenging problems such as complex field backgrounds, occlusion and small-target recognition. Overall, most agricultural YOLO research remains at the algorithm validation stage, with limited actual hardware deployment, which implies that there is still a large gap between academic research and practical field applications.

## 3. YOLO-Based Agricultural Robot Architecture

**Figure 3:**
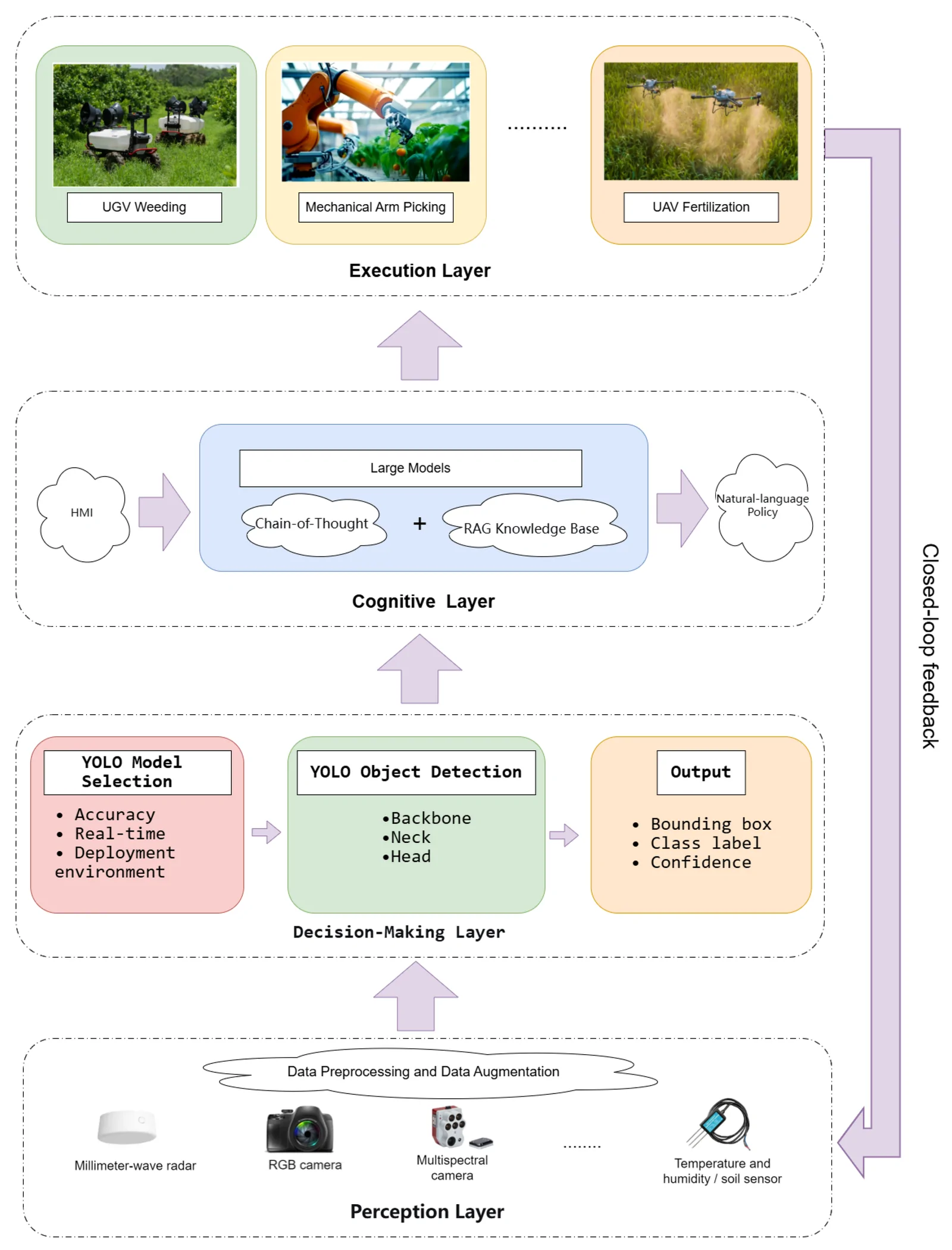
The YOLO-based agricultural robot architecture.

As shown in Figure 3, the YOLO-based agricultural robot architecture consists of four layers: the perception layer, the decision-making layer, the cognitive layer, and the execution layer. The perception layer is responsible for multimodal data collection and preprocessing; the decision-making layer performs YOLO model selection and object detection; the cognitive layer integrates large language or vision–language models for semantic reasoning; the execution layer converts decisions into control commands for actuators. The closed-loop feedback arrow in Figure 3 indicates that state information from sensors and actuators is returned to the perception layer, forming a complete perception–decision–cognition–execution loop.

### 3.1. Perception Layer

#### 3.1.1. Data Collection

The data collection stage of an agricultural robot vision system requires a complete multi-source perception system. This system typically adopts a multi-sensor collaborative architecture, including high-resolution Red-Green-Blue (RGB) industrial cameras, multispectral/hyperspectral imaging devices, 3D perception sensors, and precise positioning modules [22,23,24].

To obtain richer crop characteristic information, the system also needs to be equipped with professional spectral imaging equipment. Multispectral cameras are usually equipped with 5–12 working bands, covering the spectral range of 400–1000 nm, which can effectively distinguish crops from weeds, detect early diseases, etc. [24].

In terms of three-dimensional perception, solid-state Light Detection and Ranging (LiDAR) has become the preferred solution due to its stable performance, which can accurately obtain three-dimensional structural information of farmland scenes [25]. Meanwhile, depth cameras based on Time of Flight (ToF) or structured light principles can provide supplementary close-up 3D data. To ensure the spatial accuracy of data collection, the system also needs to integrate Real-Time Kinematic Global Navigation Satellite System (centimeter-level accuracy) and an inertial measurement unit to achieve precise spatiotemporal calibration [26].

The data collection platform supports three typical deployment modes: the mobile robot platform adopts an omnidirectional mobile chassis, which has good field passability; the drone platform is suitable for large-scale rapid monitoring; and fixed monitoring systems are used for long-term fixed-point observation. In practical applications, adaptive parameter adjustment is required for different lighting conditions and weather influences, while collection strategies should be optimized based on the complex background characteristics of farmland scenes to ensure the acquisition of high-quality basic data [27].

#### 3.1.2. Data Preprocessing and Enhancement

In the vision system of agricultural robots, data preprocessing and enhancement are key steps to ensure model performance.

Image preprocessing mainly includes techniques such as brightness/contrast adjustment, denoising, and distortion correction, which can effectively improve the quality of the original image and eliminate interference caused by environmental factors.

Data augmentation techniques expand training samples and improve model generalization ability through geometric transformations such as rotation, flipping, and scaling, as well as synthetic data generation. Geometric transformations can simulate the different postures and perspective changes of crops in natural environments [26], while synthetic data generation techniques based on Generative Adversarial Networks (GANs) can effectively solve the problem of insufficient samples of specific pests and diseases [28]. These preprocessing and enhancement methods provide more reliable input data for subsequent object detection and classification tasks.

### 3.2. Decision-Making Layer

The decision-making layer is the core of the YOLO-based agricultural robot architecture. Its main task is to process preprocessed multimodal data and generate structured detection outputs. It includes YOLO model selection, object detection, and output generation.

Model selection requires the comprehensive consideration of detection accuracy, real-time requirements, and deployment environment. Lightweight YOLO variants are preferred for real-time weed control, while models with efficient feature extraction and continuous tracking capability are more suitable for livestock behavior monitoring [29,30].

The YOLO detection network consists of a backbone for feature extraction, a neck for multi-scale feature fusion, and a head for bounding-box and class prediction. This architecture has evolved from YOLOv1 to YOLOv11, with each generation improving accuracy, speed, or deployment flexibility.

The output of the YOLO detection layer usually includes target bounding boxes, category labels, and confidence scores, which provide a quantifiable basis for downstream decision-making. The spatial information of the bounding box guides the planning of the picking path or the positioning of the spraying coordinates. Category labels are used for crop state classification, while confidence is used for result reliability evaluation and multi-model result fusion.

### 3.3. Cognitive Layer

The cognitive layer integrates large language models and vision–language models to interpret YOLO detection results and generate high-level task policies. It includes chain-of-thought reasoning, a Retrieval-Augmented Generation (RAG) knowledge base, natural-language policy generation, and a Human–Machine Interface (HMI).

Chain-of-thought reasoning enables the model to decompose complex agricultural tasks into sequential decisions, while the RAG knowledge base provides domain-specific agronomic knowledge to support reasoning. The generated natural-language policy is then passed to the execution layer as control instructions. The HMI allows human operators to monitor and intervene when necessary.

### 3.4. Execution Layer

The execution layer converts structured perception data and task decisions into physical actions. It includes motion control for the chassis and steering gear, nozzle control with PWM variable flow regulation, attitude adjustment with self-adaptive nozzle angle, and state feedback from sensors and actuators.

In precision spraying applications, the system integrates the weed spatial distribution information provided by the YOLO detection layer with the nozzle dynamics model to construct a closed-loop control system. The spraying decision not only considers target location information but also combines multidimensional factors such as crop growth stage and environmental humidity to form a dynamic spraying strategy [31].

The core of an automated harvesting system lies in the collaborative optimization of robotic arm motion planning and visual servo control. The target pose estimation provided by the YOLO detection layer provides an initial solution space for motion planning algorithms. The system needs to address the coupling problem between visual measurement delay and robotic arm dynamics constraints, which is typically achieved through predictive control frameworks or state observers [32,33]. When dealing with fruit occlusion, a multi-hypothesis tracking algorithm is adopted to maintain target state estimation [34].

The design of the autonomous navigation system integrates visual real-time positioning and mapping (V-SLAM) technology and dynamic path planning. The YOLO detection results not only are used for obstacle recognition but also enhance the hierarchical understanding of the environment through semantic segmentation [35]. Such systems typically adopt a hierarchical control strategy: the upper layer performs global path planning based on probability graphs, and the lower layer processes local obstacle avoidance through model predictive control. The uncertainty caused by environmental changes is compensated for through robust control theory or adaptive filtering methods [36].

The transition from perception to execution is not a simple sequential process. The YOLO detection output is first passed to the decision-making layer, which performs target selection, path planning, or task scheduling. The cognitive layer then interprets the detection results using large language or vision–language models, generating natural-language policies. These policies are converted into control commands for actuators such as spray nozzles, robotic arms, or mobile platforms. This perception–decision–cognition–execution chain introduces additional latency and error sources that are not captured by mAP or FPS alone, including image acquisition delay, inference latency, coordinate transformation error, communication delay, and actuator response time. For closed-loop agricultural robots, detection accuracy alone is insufficient. Key robot-specific metrics include end-to-end latency from image capture to control command output, localization error between the detection box center and the actual target center, control frequency in detection–decision–execution cycles per second, actuator response latency, and task success rate.

Finally, as indicated by the closed-loop feedback arrow in Figure 3, the state feedback from sensors and actuators is returned to the perception layer, forming a complete perception–decision–cognition–execution loop.

However, these metrics are rarely reported in most existing agricultural YOLO studies. Table 3 summarizes the non-visual indicators reported in six representative agricultural robot studies that have been deployed on actual robotic platforms.

As shown in Table 3, the reporting of non-visual indicators is highly uneven. Control frequency is not reported in any of the six studies, and localization error is reported in only one [37]. End-to-end latency is reported in three studies but with inconsistent definitions: Mehra et al. [38] measure image capture to nozzle opening, Bajraktari et al. [39] measure per-frame inference plus tracking, and Chen et al. [42] measure the complete picking cycle. Task success rate is the most reported indicator, yet its definition still varies across studies, preventing direct comparison. This imbalance shows that current research still prioritizes detection accuracy and task-level outcomes over latency, localization, and control frequency, which are decisive for real-time closed-loop operation. Future work should therefore report these robot-specific indicators alongside detection metrics and work toward standardized closed-loop benchmarks for agricultural robots.

## 4. Overview of YOLO Algorithm

### 4.1. Object Detection Algorithm

The object detection algorithm is a technology used in computer vision to recognize objects in images or videos and locate their positions. This process requires both categorizing the objects and pinpointing their location with bounding boxes. Traditional methods such as Viola Jones [43] use manual features for detection, while with the rise of deep learning, object detection algorithms are mainly divided into two categories: two-stage and single-stage. Two-stage algorithms (such as the Region-based Convolutional Neural Network (R-CNN) series [44]) generate candidate regions for reclassification and regression, with high accuracy but slow speed; single stage algorithms such as Single Shot MultiBox Detector (SSD) [45] and RetinaNet [46] directly predict the target location and category in the network, which is faster but slightly less accurate. In recent years, Transformer structures have also been introduced into the field of object detection, and new methods such as DEtection TRansformer (DETR) [47] have emerged. With just a single forward calculation, the YOLO algorithm can simultaneously predict the categories and positions of multiple targets, demonstrating significant advantages in speed and efficiency. These capabilities have made it one of the most influential algorithms in the field of real-time object detection [4].

### 4.2. Different Versions and Improvements of YOLO Algorithm

**Figure 4:**
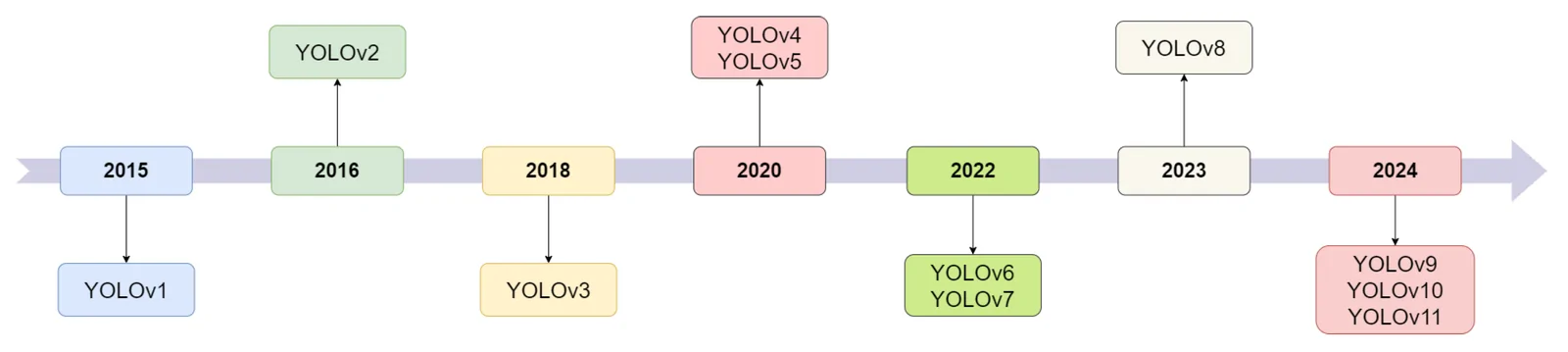
Evolution of YOLO object detection models (2015–2024).

Since its first proposal in 2015 [4], the YOLO series algorithm has become a benchmark for object detection in the field of computer vision. Figure 4 showcases the evolution of the YOLO object detection models, beginning with YOLOv1 in 2015, followed by YOLOv2, YOLOv3, YOLOv4, and other versions, culminating in the latest YOLOv11, as well as derivative versions like Scaled-YOLOv4 and YOLOX. The arrangement of these models on the timeline demonstrates the ongoing advancements and innovations of the YOLO series in the field of object detection. Table 4 shows the advantages and disadvantages of each YOLO algorithm version. The rest of this section will provide a detailed description of each section.

#### 4.2.1. YOLOv1

**Figure 5:**
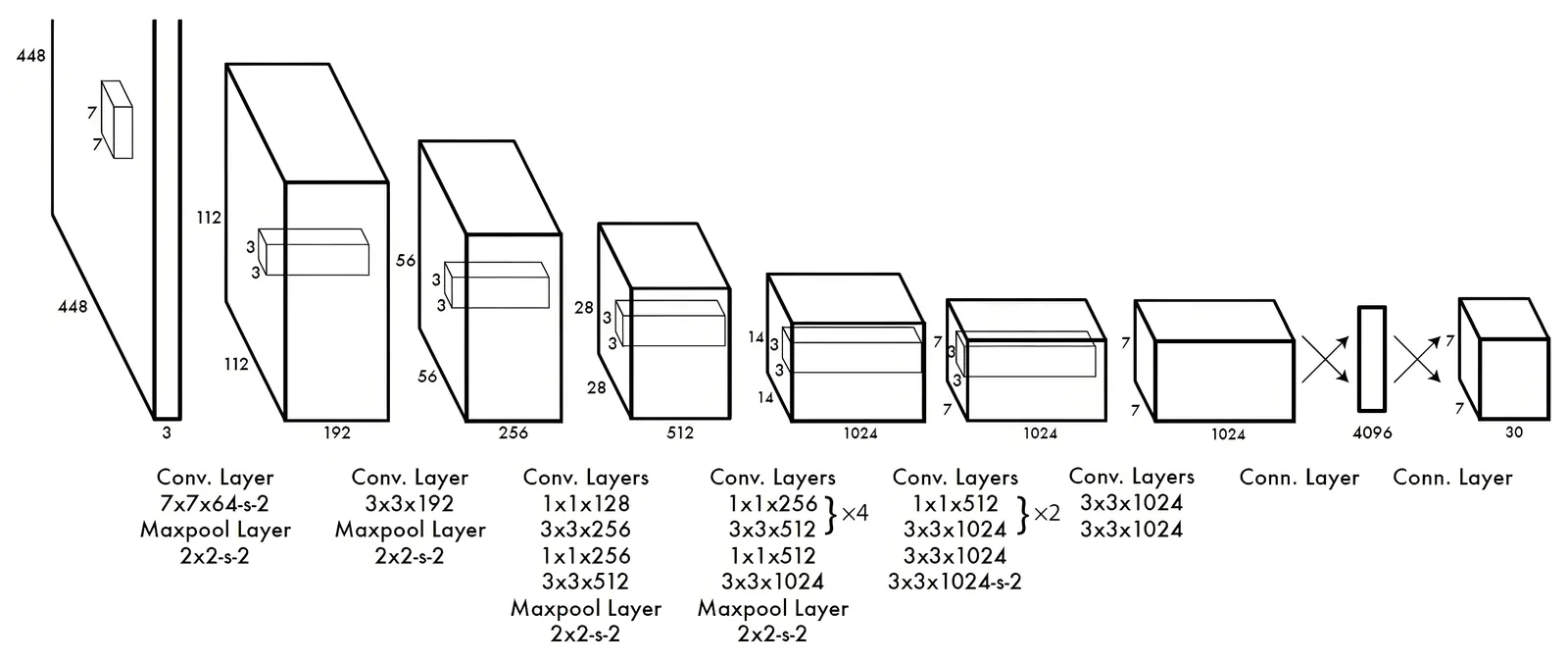
The architecture of YOLOv1.

The innovative aspect of YOLOv1 lies in simplifying the object detection task into a single-stage regression problem, as shown in Figure 5. By dividing the image into an S × S grid and predicting B bounding boxes and class probabilities for each grid cell, YOLO achieved near real-time detection speed. On the Pattern Analysis Statistical Modeling and Computational Learning Visual Object Classes (PASCAL VOC) 2007 dataset, YOLOv1 reached a mean average precision (mAP) of 63.4%, but the recall rate was only 81%, showing limitations in detecting small and overlapping objects [4,48]. In agricultural robot applications, YOLOv1, due to its lower detection accuracy, was mainly used for proof-of-concept studies in early agricultural object recognition, such as crop row detection and simple fruit localization, and has not yet been deployed in real robotic systems for real-time operation.

#### 4.2.2. YOLOv2

Building upon YOLOv1, YOLOv2 (YOLO9000) introduced a number of improvements, including Batch Normalization, which not only accelerated the model’s convergence but also increased the mAP by approximately 2% [49]. By training a high-resolution classifier at a resolution of 448 × 448, YOLOv2 enhanced its mAP by about 4% [49,50]. Furthermore, YOLOv2 employed Darknet-19 as its backbone network, as shown in Table 5, and utilized k-means clustering to automatically determine the sizes of anchor boxes, reducing the need for manual adjustments. On the COCO dataset, YOLOv2 achieved an mAP of 78.6%, with a recall rate reaching 88% [49].

**Table 4:** Analysis and comparison of different versions of the YOLO algorithm.

YOLO Version	Advantages		Disadvantages	
YOLOv1 [4]	Directly regress from images to detection results, with low computational complexity and real-time capability. 24 conv + 2 FC layers, easy to implement and train. Better foreground/background differentiation, fewer errors.		Weak feature extraction, relatively low mAP. Grid division misses small targets. Global pooling loses spatial info, harming localization.	
YOLOv2 [49]	Anchor Boxes improve localization and multi-scale detection. Batch Normalization speeds convergence, reduces overfitting. Multi-scale training improves adaptability.		Weak performance on very small objects. Loss function too simple for small target detection.	
YOLOv3 [51]	Darknet-53 backbone, stronger feature extraction. Multi-scale prediction improves small object detection.		Slightly slower due to multi-scale fusion. More complex, higher resource demand.	
YOLOv4 [8]	Enhanced structure/modules improve accuracy. Real-time and edge-device friendly.		Needs strong GPU to keep speed.	
YOLOv5	High accuracy with fast FP16 inference. Different model sizes (n, s, m, l, x) for flexibility.		Unofficial, lacking standardization. Sometimes slightly less accurate than YOLOv4.	
YOLOv6 [52]	Optimized for industry, fast inference. 47.9% mAP and 80 FPS on COCO.		Weak in overlapping objects.	
YOLOv7 [53]	Improved structure, loss, and sample matching. Dynamic label allocation aids training.		Complex network, higher computation. Still hard for small/dense targets.	
YOLOv8 [54]	Transformer+CNN with multi-scale fusion. Anchor-free detection increases flexibility.		Higher complexity and hardware demand.	
YOLOv9 [55]	PGI + GELAN, better accuracy/efficiency.		Needs more training resources. Complex, unsuitable for low-resource devices.	
YOLOv10 [14]	Multi-scale fusion improves detection. Dynamic anchor adapts to inputs.		Still weak in extreme small/dense targets. Strongly data-quality dependent.	
YOLOv11 [15]	New components (C3k2, SPPF, C2PSA) enhance features. Supports detection + multiple CV tasks.		Higher computation. Longer training/inference, harder tuning.	
**YOLO Version**	**Dataset**	**Metric**	**Speed**	**Hardware**
YOLOv1 [4]	PASCAL VOC 2007	mAP = 63.4%, Recall = 81%	Not specified	CPU
YOLOv2 [49]	COCO	mAP = 78.6%, Recall = 88%	Not specified	GPU
YOLOv3 [51]	COCO	mAP = 57.9%	40 FPS	GPU
YOLOv4 [8]	COCO	mAP = 43.5%	65 FPS	GPU
YOLOv5	COCO	mAP = 49.4%	60 FPS	High-end GPU
YOLOv6 [52]	COCO	mAP = 45.5%	70–80 FPS	Industrial GPU
YOLOv7 [53]	COCO	mAP = 50.5%	80 FPS	High-end GPU
YOLOv8 [54]	COCO	YOLOv8x mAP = 53.9%	Input 640 px	GPU
YOLOv9 [55]	MS COCO	mAP = 55.6%	Not specified	High-end GPU
YOLOv10 [14]	COCO	mAP = 54.4%	Low-latency	GPU
YOLOv11 [15]	Not specified	Not specified	Not specified	GPU

Note: The values above are reported from each respective original paper under different experimental conditions (dataset versions, input resolutions, hardware platforms, and evaluation protocols). Direct cross-version comparison of absolute values is not recommended; comparisons should focus on relative improvement trends and architectural design characteristics.

**Table 5:** Darknet-19 framework.

Type	Filters	Size/Stride	Output
Convolutional	32	3 × 3	224 × 224
Maxpool	2 × 2/2		112 × 112
Convolutional	64	3 × 3	112 × 112
Maxpool	2 × 2/2		56 × 56
Convolutional	128	3 × 3	56 × 56
Convolutional	64	1 × 1	56 × 56
Convolutional	128	3 × 3	56 × 56
Maxpool	2 × 2/2		28 × 28
Convolutional	256	3 × 3	28 × 28
Convolutional	128	1 × 1	28 × 28
Convolutional	256	3 × 3	28 × 28
Maxpool	2 × 2/2		14 × 14
Convolutional	512	3 × 3	14 × 14
Convolutional	256	1 × 1	14 × 14
Convolutional	512	3 × 3	14 × 14
Convolutional	256	1 × 1	14 × 14
Convolutional	512	3 × 3	14 × 14
Maxpool	2 × 2/2		7 × 7
Convolutional	1024	3 × 3	7 × 7
Convolutional	512	1 × 1	7 × 7
Convolutional	1024	3 × 3	7 × 7
Convolutional	512	1 × 1	7 × 7
Convolutional	1024	3 × 3	7 × 7
Convolutional	1000	1 × 1	7 × 7
Avgpool Softmax	Global		1000

#### 4.2.3. YOLOv3

**Figure 6:**
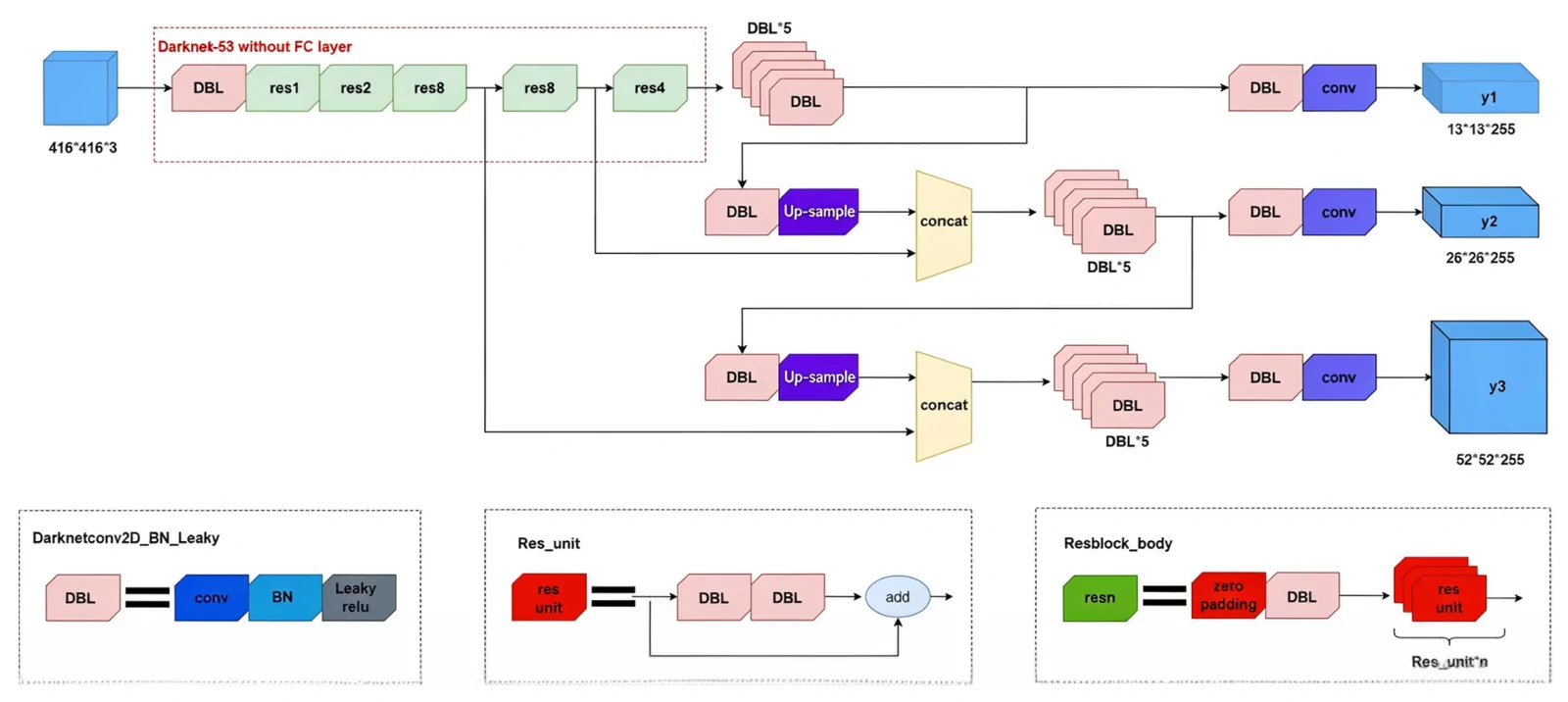
Multi-scale detection architecture.

The introduction of YOLOv3 in 2018 by Joesph Redmon et al. [51]. YOLOv3 further optimized the network structure by introducing multi-scale feature fusion, as shown in Figure 6, using three different scale feature maps (13 × 13, 26 × 26, 52 × 52) to improve the detection ability for small targets. YOLOv3 achieved an mAP of 57.9% and a frame rate of 40 FPS on the COCO dataset [56]. In addition, YOLOv3 also introduces Darknet-53 as the backbone network and enhances feature extraction capabilities through cross layer connections and residual connections [51]. In agricultural robot applications, YOLOv3’s multi-scale prediction capability enables simultaneous detection of large-scale crops and small-scale weeds and pests, and has been widely used in UAV weed spraying systems [6,7] and visual guidance for orchard fruit harvesting robots.

#### 4.2.4. YOLOv4

**Figure 7:**
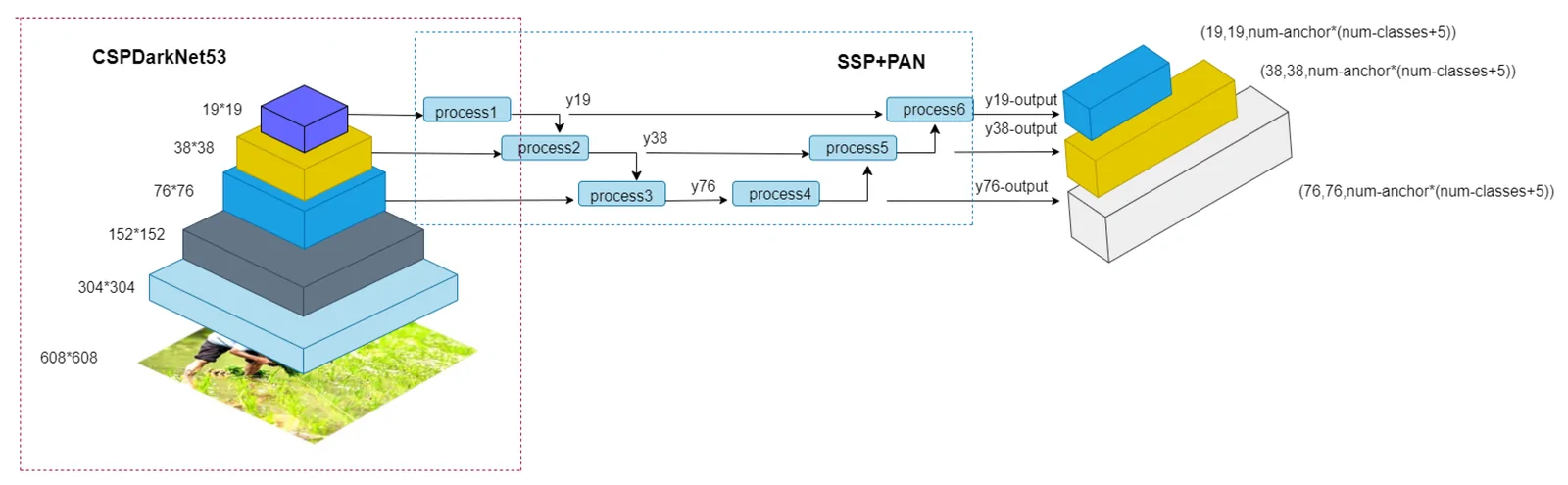
Architecture diagram of YOLOv4.

In April 2020, a team led by Alexey Bochkovskiy introduced YOLOv4, representing a profound departure from its predecessors with revolutionary architectural changes aimed at improving performance while maintaining real-time capabilities [8]. Building on YOLOv3, YOLOv4 integrated a variety of the latest computer vision technologies, such as the CSPDarkNet53 backbone network, SPP and PAN networks, and the Mish activation function [8,57], further enhancing the model’s performance, as shown in Figure 7. YOLOv4 achieved an mAP of 43.5% on the COCO dataset while maintaining a high frame rate of 65 frames per second (FPS) [58].

#### 4.2.5. YOLOv5

In 2020, Glenn Jocher introduced YOLOv5, following the release of YOLOv4. YOLOv5 has optimized the model design, offering various scaled models including YOLOv5s, YOLOv5m, YOLOv5l, and YOLOv5x [9]. The main differences among them lie in the model size, number of parameters, and average precision, as shown in Table 6. By introducing CSPNet and CSPPAN structures, these architectural improvements effectively enhance the model’s detection speed and accuracy [59,60]. These structures maintain network performance while reducing computational load, achieving a good balance between speed and accuracy. On the COCO dataset, YOLOv5 achieved an mAP of 49.4%, with a frame rate as high as 60 FPS, indicating the model’s ability to quickly process video streams, and making the model suitable for real-time object detection tasks [3]. In addition, YOLOv5 supports various optimization techniques, such as model pruning and quantization, further enhancing the model’s deployment efficiency. In agricultural robots, YOLOv5 has become the mainstream choice for agricultural robot vision systems due to its modular design and multiple model size options, applied in weeding robots [29], fruit counting and yield estimation [61], and livestock behavior monitoring [30].

#### 4.2.6. YOLOv6

**Figure 8:**
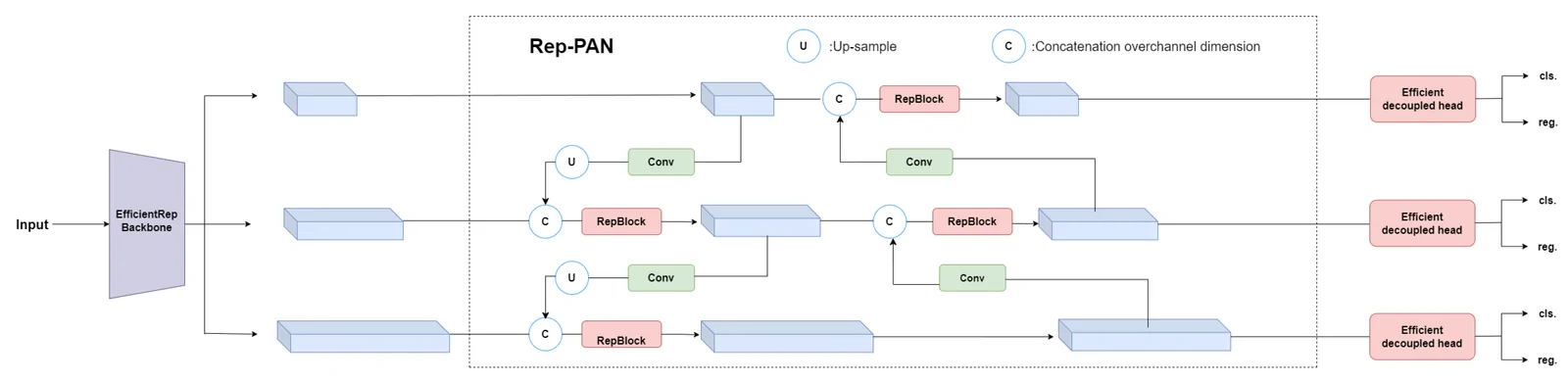
Architecture diagram of YOLOv6.

Released in September 2022 by the Meituan Vision AI Department [52], YOLOv6 is a single-stage object detection framework designed speciffcally for industrial applications. YOLOv6 is designed with industrial applications in mind, focusing on optimizing the model for deployment in resource-constrained environments. The architecture utilizes the efficiency and flexibility renowned RepVGG as part of the backbone network, and introduces some concepts of Cross Stage Partial Network (CSP-Net), such as the CSPStackRep block, in subsequent versions to further improve computational efficiency [52,62], as shown in Figure 8. This combination allows YOLOv6 to deliver high performance while maintaining low computational overhead. On the COCO dataset, a benchmark for object detection models, YOLOv6 achieves an impressive mAP of 45.5%, indicating the model’s accuracy in detecting objects across various categories [5]. Moreover, the algorithm’s frame rate of 70 FPS demonstrates the model’s capability for real-time processing, which is crucial for applications like surveillance, robotics, and autonomous vehicles that require instant feedback and decision-making based on visual input [52,63]. These advancements make YOLOv6 a strong contender for practical use cases where both accuracy and speed are paramount.

#### 4.2.7. YOLOv7

**Figure 9:**
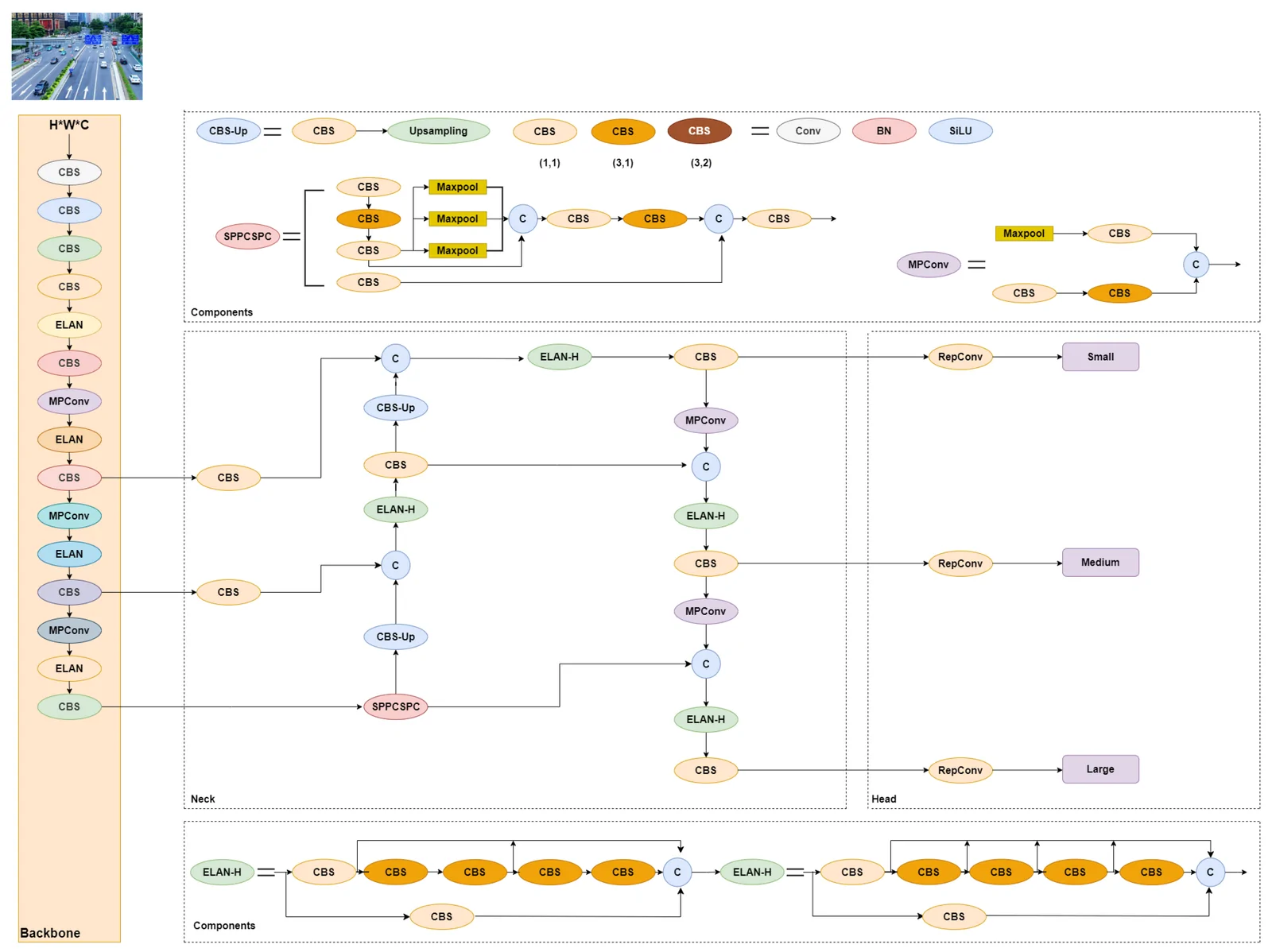
Architecture diagram of YOLOv7.

Introduced in July 2022, YOLOv7 marked a significant leap forward from previous YOLO versions, showcasing improved accuracy and speed enhancements ranging from 5 FPS to 160 FPS [53,64]. YOLOv7 has introduced innovative architecture and training strategies in the field of object detection, significantly enhancing the model’s efficiency and accuracy shown in Figure 9. This model is entirely trained from scratch on the COCO dataset, without the use of other datasets or pre-trained weights, which is quite rare for real-time object detectors. YOLOv7 has achieved an mAP score of 50.5% on the COCO dataset while maintaining a high frame rate of 80 FPS, demonstrating that the model can achieve impressive accuracy while maintaining high speed [53]. Additionally, YOLOv7 has introduced trainable auxiliary architectures, such as YOLOR and RepVGG, which enhance the model’s precision by increasing the training cost without adding to the inference cost [53]. YOLOv7 has also adopted models related to a variety of computer vision tasks, further enhancing the model’s application capabilities in different scenarios. Overall, YOLOv7 has set a new benchmark in the field of object detection through the model’s advanced architectural design and training strategies.

#### 4.2.8. YOLOv8

YOLOv8, a significant product launched by Ultralytics in January 2023 [54,65], marks a notable advancement in the field of computer vision, particularly in real-time object detection. This latest iteration of the YOLO series boasts an impressive precision rate of 97.1%, enhancing reliability and reducing false positives in practical applications [66]. YOLOv8 adopts an anchor-free detection paradigm, simplifying the training process and improving NMS efficiency. The model no longer relies on pre-defined anchor boxes but directly predicts target centers and bounding box offsets. YOLOv8 introduces a Task-Aligned Assigner sample assignment strategy that assigns positive samples based on a joint metric of classification and localization, replacing the adaptive anchor box selection used in YOLOv5. YOLOv8 is offered in five variants, each tailored to specific accuracy and computational requirements, providing a versatile solution for a wide range of object detection tasks, as detailed in Table 7.

#### 4.2.9. YOLOv9

YOLOv9, released in February 2024 represents the latest addition to the mainstream YOLO variants [55]. YOLOv9 has significantly improved the model’s performance in object detection tasks by introducing Programmable Gradient Information (PGI) technology. PGI optimizes the propagation of gradient information through auxiliary reversible branches and multi-level auxiliary information, enhancing the model’s interpretability, robustness, and versatility across various scenarios. On the MS COCO dataset, YOLOv9 achieved an impressive object detection performance with an mAP of 55.6%, demonstrating the model’s competitiveness in the field of real-time object detection [55,67].

#### 4.2.10. YOLOv10

Building upon the momentum of innovation in the YOLO series, another groundbreaking development emerged in the same year with the release of YOLOv10, also in 2024 [14]. YOLOv10 achieved NMS-free training through a consistent dual assignment strategy that considers both NMS-related and NMS-agnostic label assignments during training, eliminating the need for NMS post-processing during inference and thereby reducing inference latency [14,19]. These enhancements have propelled YOLOv10 to achieve an mAP of 54.4% on the COCO dataset while maintaining low latency, demonstrating the powerful capabilities of the YOLO series in real-time object detection [14]. The design of YOLOv10 optimizes parameter utilization efficiency, leading to notable improvements in performance and efficiency across various model scales.

#### 4.2.11. YOLOv11

YOLOv11 is the latest version in the YOLO series [15], featuring significant enhancements in the architecture of object detection models, including the introduction of innovative components such as the C3k2 block, SPPF, and C2PSA, which contribute to improving model performance, especially in terms of enhanced feature extraction. While keeping the number of parameters compact, YOLOv11 has increased accuracy and achieved a significant boost in processing speed, thus making substantial progress in real-time performance capabilities. Additionally, YOLOv11 demonstrates the model’s expanded capabilities across a variety of computer vision tasks, including object detection, instance segmentation, pose estimation, and Oriented Object Detection (OOD), and shows exceptional versatility across different model sizes (from nano to extra-large), meeting the diverse application needs from edge devices to high-performance computing environments. Overall, YOLOv11 has become an important advancement in real-time computer vision applications, balancing accuracy, computational efficiency, and multi-task capabilities.

Examining the architectural evolution of the YOLO series from the perspective of agricultural robot applications reveals several key trends that are particularly relevant to agricultural robotics. First, the accuracy–speed–energy trade-off has gradually shifted from merely pursuing higher mAP to balancing detection performance with inference latency and power consumption. YOLOv5’s multi-size design and YOLOv10’s NMS-free strategy are especially suitable for battery-powered agricultural robots, whereas deeper architectures such as YOLOv9 require substantial computational resources and have lower deployment feasibility on edge devices. Second, small-object detection remains a critical challenge since many agricultural targets (e.g., weed seedlings, small fruit buds, and early disease lesions) occupy only a few pixels. The multi-scale detection head in YOLOv5 and the anchor-free design in YOLOv8 have improved this situation, but the performance degradation on high-resolution UAV imagery and under severe occlusion is still not fully resolved. Third, robustness under field conditions is essential. Complex backgrounds, illumination variations, soil interference, and canopy occlusion can significantly reduce detection accuracy, and most reported results are still obtained under laboratory or offline conditions, which means actual field performance may be lower. Fourth, suitability for resource-constrained hardware is a decisive factor for agricultural robots. Lightweight variants such as YOLOv5n and YOLOv8n, together with NMS-free designs, offer clear advantages for edge deployment.

Compared with other detection frameworks, the YOLO series provides the most favorable balance among accuracy, speed, energy efficiency, and deployment flexibility. Two-stage detectors such as Faster R-CNN remain useful for high-precision offline analysis but are too slow for real-time field operations, while DETR-based methods suffer from long training convergence and insufficient small-object performance on edge devices. These observations indicate that future YOLO development for agricultural robotics should focus not only on accuracy improvement but also on energy efficiency, field robustness, and edge adaptability.

### 4.3. Object Detection Algorithms Evaluation Method

#### 4.3.1. Comparative Analysis of Object Detection Algorithms

Different algorithms exhibit unique performance characteristics in object detection tasks, which stem from differences in algorithm design concepts, model architectures, and implementation strategies. The YOLO series algorithm adopts a single-stage detection architecture, which has high real-time performance, but the detection accuracy is limited due to the loss of small target feature information caused by downsampling operations [53]. DETR implements end-to-end detection based on Transformer, but the global attention mechanism has high computational complexity and requires high hardware computing power [47]. Two-stage detectors such as Faster R-CNN improve accuracy through region proposal networks, but their inference speed is slow, making it difficult to meet the real-time requirements [68]. Therefore, algorithm selection needs to be based on actual needs, balancing accuracy, speed, and computational cost. Real-time scenarios may lean towards YOLO, and high-precision tasks may choose two-stage methods, while DETR is more suitable for applications that require end-to-end detection.

#### 4.3.2. Evaluation Metrics for Object Detection Algorithm

Against the backdrop of rapid development in smart agriculture, deep learning object detection algorithms have become the core technology for crop monitoring, disease and pest identification, and agricultural automation equipment. However, the particularity of agricultural scenes, such as complex backgrounds, variable target scales, and unstable lighting conditions, poses higher requirements for algorithm evaluation. This article systematically summarizes the key evaluation indicators for target detection in the agricultural field. These metrics cover accuracy, precision, recall, mAP, intersection over union (IoU), and detection speed (FPS) [20,69]. Through a comprehensive review and in-depth interpretation of various evaluation indicators, the aim is to provide a clear decision-making basis for academia and industry, assist them in accurately matching the optimal object detection algorithm in diverse scenarios, and continuously promote the improvement of model performance. As shown in Table 8, the calculation formulas for the six indicators mentioned above are as follows:

(1) $$Accuracy=\frac{TP+TN}{TP+TN+FP+FN}$$

Among them, true positive (TP) represents the number of positive-class samples correctly identified by the model; True Negative (TN) represents the number of negative-class samples correctly identified by the model; false positive (FP) refers to the number of samples in which the model incorrectly predicts a negative class as a positive class; and false negative (FN) refers to the number of positive-class samples missed by the model.

(2) $$Precision=\frac{TP}{TP+FP}$$

(3) $$Recall=\frac{TP}{TP+FN}$$

(4) $$AP=\int_{0}^{1}P\left(r\right)\;dr$$

(5) $$mAP=\frac{1}{C}\sum_{i=1}^{C}{AP}_{i}$$

By adjusting the confidence threshold of the detection box output by the model, the system will provide different numbers of TP, FP, FN, thus obtaining a series of (recall and precision) points. Connect these points in ascending recall order to obtain a PR curve. Calculate the area under the curve using the trapezoidal or integral method to obtain the AP value of the category within the 0–1 recall range. The larger the area, the better the detection performance:

(6) $$IoU=\frac{Prediction\cap GroundTruth}{Prediction\cup GroundTruth}$$

GroundTruth is the true bounding box area of the actual target; prediction is the target bounding box region predicted by the model.

(7) $$FPS=\frac{Frames}{Times}$$

Frames is the total number of image frames successfully processed by the model during testing. Times is the total time required to complete all the frame processing mentioned above.

### 4.4. Key Issues to Be Addressed for YOLO Detection System

Although YOLO-based object detection systems have excellent performance in real-time and efficiency, they still face several key challenges in practical applications, including the balance between detection accuracy and speed, limitations in small object detection, insufficient robustness in complex scenes, and unstable performance of model generalization ability under different data distributions. This section will discuss the significance of small object detection problems and the balance between accuracy and speed, and provide solutions as shown in Table 9.

#### 4.4.1. Small Object Detection Problem

Small object detection has always been a highly challenging problem in object detection tasks. This is mainly due to two key factors: Firstly, small targets occupy a limited pixel area in the image, and after multiple downsampling, their feature information is easily lost in the deep network; Secondly, small targets often have complex situations such as occlusion and lighting changes, further increasing the difficulty of detection. Taking the YOLO series algorithm as an example, the algorithm’s default input resolution of 640 × 640 will excessively compress the feature information of small targets in the backbone network, resulting in a significant increase in missed detection rate.

Researchers have proposed various innovative solutions to address this issue. An effective method is to enhance the feature representation ability of small targets by improving the feature pyramid structure. For example, the Feature Pyramid Network (FPN) proposed by research team. Significantly improves the detection performance of small targets by constructing a top–down feature fusion path that combines high-level semantic information with low-level detail features [70]. Experiments have shown that in the COCO dataset, the FPN structure can increase the AP of small targets by about 5%. Another solution is to adopt a high-resolution input strategy. A composite scaling method was proposed in YOLOv7, which dynamically adjusts the input resolution to preserve the detailed features of small targets [53]. When the input resolution is increased from 640 × 640 to 1280 × 1280, the recall rate of small targets can be increased by more than 15%, which verifies the key impact of resolution on small target detection. These methods not only solve the problem of feature loss in small object detection but also provide new optimization ideas for object detection in complex scenes.

#### 4.4.2. The Balance Between Accuracy and Speed

In the field of object detection, mAP and FPS have always been core challenges, and this contradiction mainly stems from two aspects: on the one hand, improving detection accuracy usually requires deeper network structures and larger input resolutions, which inevitably increases computational burden; On the other hand, the pursuit of real-time performance forces the model to compress computational complexity, resulting in a decrease in feature extraction capability. Although the single-stage detectors represented by the YOLO series have advantages in speed, they still face the problem of sudden drops in accuracy when dealing with complex scenes.

Researchers have proposed various innovative solutions to address this contradiction. MobileNetV2 significantly reduces computational complexity while maintaining high accuracy by introducing inverse residual structures and linear bottlenecks [72]. This approach achieves a top-1 accuracy of 72% in ImageNet classification tasks with only 300M multiply add operations, providing an important paradigm for lightweight network design. Another breakthrough idea comes from model quantization technology. An integer quantization method was proposed to convert a 32-bit floating point model into an 8-bit integer model [73], which makes the reasoning speed of ResNet50 three times faster without losing precision. This technology has been widely used in edge computing equipment. These methods not only solve the trade-off between accuracy and speed, but also promote the industrial level implementation of object detection technology, pointing out the optimization direction for subsequent research.

### 4.5. YOLO-Based Object Detection Model in Agricultural Robots

With the development of agricultural intelligence, a single visual algorithm is no longer sufficient to meet the needs of complex farmland scenes. As an efficient real-time detection framework, YOLO performs well in speed and accuracy, but there are still limitations in small object detection, occlusion processing, and multimodal data fusion. Therefore, researchers have combined YOLO with other technologies to enhance the environmental perception and decision-making capabilities of agricultural robots. Table 10 shows different YOLO object detection models.

The YOLO-based object detection model has shown significant advantages when combined with other technologies, effectively breaking through the limitations of a single visual algorithm. For example, in the field of infrared and visible light image fusion, the Dual YOLO architecture significantly improves the accuracy of infrared target detection by integrating YOLOv7’s dual feature extraction channels, combining attention fusion module and Inception SE module. The experiment shows that the model achieves 71.8% mAP on the DroneVehicle remote sensing dataset and 73.2% mAP on the KAIST pedestrian dataset, effectively solving the problem of false detection in infrared images due to the lack of texture information [80].

In terms of object detection in complex environments, VESY sensors integrate YOLO with saliency mapping, utilizing dehazing algorithms and stereo depth information to significantly improve detection performance under foggy conditions. This scheme achieves accurate recognition of vehicles in fog by integrating salient bounding boxes with YOLO detection results, making up for the shortcomings of traditional YOLO in low visibility environments [81]. These cases demonstrate that the combination of YOLO with multimodal data fusion, attention mechanisms, and other technologies can significantly improve detection accuracy, robustness, and real-time performance, providing a reliable solution for practical applications in the field of intelligent agriculture.

## 5. The Large Model for Agriculture

Agricultural large model refers to an AI system that is fine-tuned or trained specifically for the agricultural sector, based on large-scale language models (such as the GPT series) or multimodal models (such as models that combine image and sensor data). It can handle agricultural-related data, such as crop growth, soil analysis, and climate prediction, helping farmers, researchers, and businesses improve efficiency. Next, this section will explain the core content that needs to be covered when introducing agricultural large models from a structured perspective.

### 5.1. The Core Architecture of Agricultural Large Models

**Figure 10:**
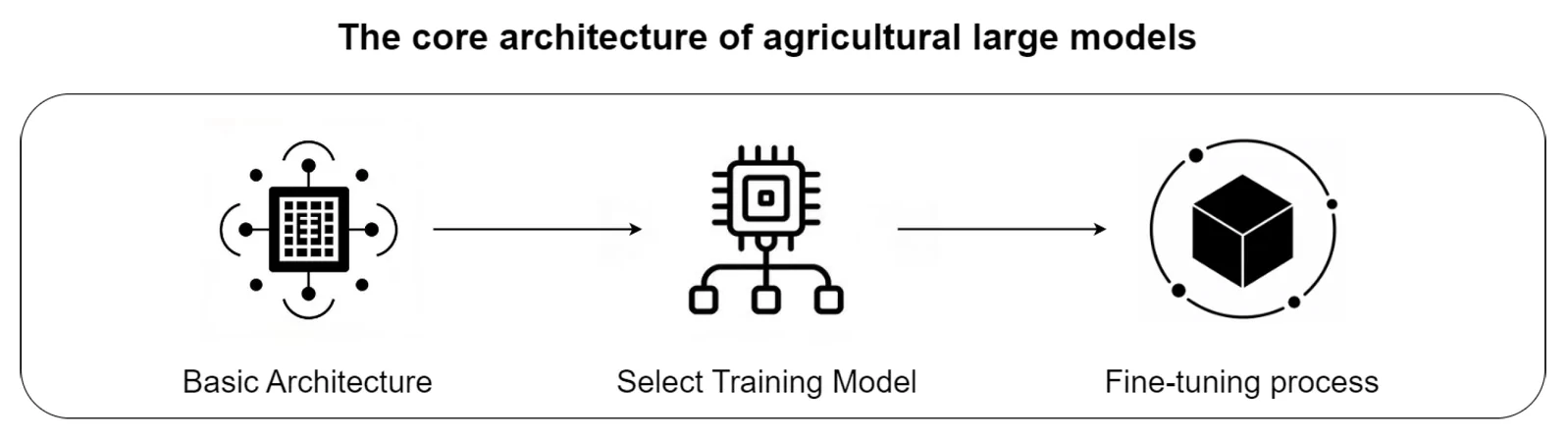
The core architecture of agricultural large models.

The core architecture of large agricultural models is typically built upon modern deep learning frameworks, particularly the Transformer architecture, which is a revolutionary neural network structure [82]. It has become the cornerstone of most large language models (LLMs), including Bidirectional Encoder Representations from Transformers (BERT) and the Llama series. Figure 10 below illustrates the core architecture of the model, encompassing the basic architecture, pre-training process, and fine-tuning mechanism.

#### 5.1.1. Basic Architecture: Transformer Architecture

Transformer is the core “skeleton” of large agricultural models. Unlike traditional RNNs, it is based on a self-attention mechanism, allowing the model to process sequence data in parallel, greatly enhancing efficiency and performance [82].

**Self-Attention:** This is the core innovation of Transformer. Simply put, it allows the model to “focus” on all relevant parts of a sequence when processing a sentence or data sequence, rather than processing them one by one. The self-attention formula is as follows:

(8) $$\mathrm{Attention}\left(Q,K,V\right)=\mathrm{softmax}\left(\frac{{\mathrm{QK}}^{⊤}}{\sqrt{d_{k}}}\right)V$$

Among them, Query (Q), Key (K), and Value (V) are linear transformations of the input vector, and $d_{k}$ is the dimension. This allows the model to capture long-distance dependencies, while agricultural data often involves spatiotemporal sequences (such as crop growth cycles).

**Multi-Head Attention:** The attention mechanism is divided into multiple “heads”, each of which performs independent computation and is then concatenated. A typical Transformer has 8–64 heads, enhancing the model’s expressive capability.

**Feed-Forward Network:** Each attention layer is followed by a fully connected layer for nonlinear transformation.

**Positional Encoding:** Because Transformers do not handle order, sine/cosine functions are added to encode positional information.

Agricultural data is multimodal and complex: text, images, and numerical values. Transformer can be easily extended to multimodal models, such as Vision Transformer for processing images or Time-Series Transformer for processing time-series data.

#### 5.1.2. Pre-Training Process

Agricultural large models are typically not trained from scratch but rather start from general pre-trained models such as BERT or the Llama series. These models are first pre-trained on a vast amount of general data and then adapted to the agricultural domain.

The BERT series, proposed by Google in 2018 [83], is innovative in that it is the first to achieve true “bidirectional” contextual modeling during the pre-training stage. It uses Masked Language Modeling (MLM) to randomly mask 15% of the words and forces the model to rely solely on information from both sides to reconstruct the original words. Simultaneously, Next Sentence Prediction (NSP) is used to determine whether two sentences are consecutive, allowing the deep Transformer encoder to learn word-level and sentence-level representations simultaneously. Pre-training was performed on a mixed corpus of Wikipedia and BooksCorpus containing 3.3 billion words, using a large batch size of 256 × 512 tokens, an Adam optimizer, and a linearly decaying learning rate. Convergence was achieved in approximately 4 days on 4–16 Cloud TPUs. Thanks to bidirectional context fusion, BERT performs excellently in downstream relation extraction tasks. For instance, on the SemEval-2010 dataset, it significantly outperforms traditional models such as CNN and RNN, achieving an F1 score improvement of over 5%.

The Llama series, open-sourced by Meta since 2023, employs a Decoder-Only Transformer architecture [84,85]. It is pre-trained in an autoregressive manner using a mixed corpus of approximately 1–2 T tokens from public web pages, books, and code. With optimizations such as Group Query Attention, Rotational Embedding, and SwiGLU activation, it achieves efficient generation within a 4096-token contextual window. This model can be directly applied to long-text generation tasks such as crop management plans or question-and-answer consultations in agricultural scenarios without requiring retraining.

#### 5.1.3. Fine-Tuning Mechanism

After pre-training, the model is fine-tuned to adapt to the agricultural domain. This is a crucial step, where typically only 1–10% of the pre-trained parameters require data, yet it can significantly enhance performance.

The fine-tuning of large agricultural models first requires the construction of a domain dataset, typically integrating multimodal data such as statistics from the United Nations Food and Agriculture Organization, satellite remote sensing images, and sensor time series (with a scale of approximately 10GB-1TB), to specifically enhance the model’s understanding of agricultural knowledge. The fine-tuning employs parameter-efficient methods (such as LoRA [86]) to reduce computational overhead, and combines supervised fine-tuning or reinforcement learning to optimize task performance; in multimodal scenarios, text and image representations can be jointly trained [87]. Frameworks such as Hugging Face [88] are commonly used in training, and performance is evaluated through metrics such as accuracy, BLEU, or mean squared error.

### 5.2. Data Source

In the training process of large agricultural models, data sources serve as the core foundation for model performance. These data not only determine the accuracy and generalization ability of the model but also directly affect its applicability in real-world agricultural scenarios. Large agricultural models typically utilize multimodal data to capture the complexity of agricultural systems, such as crop growth dynamics, environmental factors, and human interventions. Training data often originates from public databases, satellite systems, field collection, and academic resources, with a total size that can reach several terabytes or even petabytes. Data collection requires attention to diversity, quality, and privacy protection. The main data types include text data, image data, and sensor data.

#### 5.2.1. Text Data

The text data for the agricultural large model primarily originates from authoritative texts such as the annual reports of the Food and Agriculture Organization, agricultural sector statistics, professional papers, and online knowledge bases [89,90]. Its core function is to endow the model with semantic understanding and generation capabilities for agricultural terminology, industry reports, market analysis, and scientific research literature. This type of data is typically trained in the form of a masked language model (MLM) and fine-tuning language models like BERT with billions of professional corpora, enabling the model to accurately comprehend professional expressions like “NDVI index” and support downstream tasks such as crop recommendations and disaster assessments.

#### 5.2.2. Image Data

Image data supports visual perception tasks in large agricultural models. Its core sources include high-resolution field photos captured by drones (such as the PlantVillage pest and disease dataset [91]), satellite images containing multiple spectral bands (such as Sentinel-2 [92]), and crowdsourced images captured from the ground [93]. This data is trained through architectures such as Vision Transformer (ViT) [94], often combined with data augmentation to enhance model robustness. In a multimodal framework, images are paired with text descriptions for cross-modal learning, enabling the model to map visual inputs to semantic outputs, thus serving precision agriculture applications such as crop identification, disease detection, and land use classification.

#### 5.2.3. Sensor Data

Sensor data serves as structured inputs for real-time decision-making and quantitative prediction by large agricultural models. It is typically collected continuously by Internet of Things (IoT) devices or meteorological stations, forming time-series feature vectors [95]. During training, this numerical data is converted and input into models such as multi-layer perceptions or long short-term memory networks to learn complex mapping relationships between, for example, “soil moisture thresholds” and “irrigation decisions”. The scale of such data can reach millions of points, and after processing with edge computing, it provides a core basis for tasks such as precision irrigation, growth environment regulation, and yield prediction.

### 5.3. Key Technology

The key technologies of agricultural large models primarily revolve around how to efficiently process complex, multi-source agricultural data and achieve real-time, intelligent decision-making. These technologies assist the model in transitioning from “understanding” data to “action” support, significantly enhancing the level of precision agriculture. They encompass three major technologies: multimodal fusion, reinforcement learning, and edge computing.

#### 5.3.1. Multimodal Fusion

Multimodal fusion [87] is a key technology to address the multi-source and heterogeneous characteristics of agricultural data. It aims to achieve collaborative understanding and decision-making through the joint modeling of modalities such as text, images, and sensors, leveraging complementary information. A typical paradigm is the contrastive learning model represented by CLIP, which aligns images and texts in a shared semantic space to support zero/few-shot agricultural visual recognition tasks. Further innovative fusion models, such as enhanced robust CT-CLIP, or LLaVA-like architectures [96], have achieved more complex cross-modal feature interactions on this basis, applicable to scenarios such as fusing satellite imagery and meteorological reports to predict yields or monitor pests and diseases. In the agricultural field, intermediate fusion strategies are often adopted for feature-level alignment and integration, simulating human comprehensive perception capabilities and enhancing the accuracy and robustness of models in complex field environments. Nevertheless, these outcomes are mostly obtained from static offline datasets; end-to-end deployment and systematic testing within closed-loop agricultural robotic platforms under real-world farm conditions are still absent.

#### 5.3.2. Reinforcement Learning

Reinforcement learning, through its “action-reward” trial-and-error mechanism, provides agricultural large models with the ability to optimize decision sequences in uncertain environments, serving as a key technology for achieving adaptive and precise management [97]. Its typical application is to combine the knowledge generated by large models with reinforcement learning algorithms to optimize dynamic control tasks such as drone pesticide application paths, aiming to maximize benefits and minimize costs and risks [98,99]. Through human-feedback-based reinforcement learning, model outputs can be further calibrated to ensure the safety and practicality of agricultural recommendations, thereby forming an end-to-end intelligent agricultural closed loop from perception, prediction to decision execution. The integration of reinforcement learning with large models for agricultural decision-making has not yet been experimentally demonstrated in real field conditions and remains a future research direction.

#### 5.3.3. Edge Computing

Edge computing effectively addresses the challenges of poor network coverage, high latency, and data privacy sensitivity in agricultural fields by deploying compressed and optimized multimodal models on local devices such as drones and sensor gateways. Typical implementations, such as the Farm-LightSeek framework, enable the real-time fusion of image and IoT sensor data at edge nodes for disease detection and decision-making, synchronizing with the cloud only when necessary. This reduces response latency from seconds to milliseconds, enables real-time analysis in offline environments, significantly reduces bandwidth consumption, and ensures local privacy of farm data [100]. It should be noted that such frameworks are mainly validated in simulation environments, and field validation on mobile agricultural robot hardware that combines YOLO perception outputs with large-model reasoning is still lacking.

## 6. The Applications of YOLO in Agricultural Robots in Different Fields

### 6.1. Weed Detection

Weed detection is crucial for modern agriculture, as it directly affects crop yield and the efficient use of resources. Traditional weed control methods typically require a significant amount of labor and time. Integrating YOLO variants into weed detection robots provides a solution for real-time and efficient identification of weeds in farmland; Meanwhile, by integrating with large-scale models, the intelligence of the system has been further enhanced.

This section explores the application of YOLO in weed detection, focusing on the algorithm’s potential to completely transform weed management practices. We delve into the challenges faced in weed detection within traditional agriculture and examine how YOLO variants address these challenges. Furthermore, we investigate the performance of YOLO in accurately identifying and locating weeds in real-world scenarios, demonstrating the algorithm’s effectiveness in advancing the development of agriculture robots.

In terms of smart agricultural machinery guidance, the YOLO algorithm provides precise weed location information, enabling agricultural machinery to apply herbicides accurately [101]. This improves weeding efficiency and theoretically enables more precise herbicide application. In addition, the YOLO algorithm has a wide range of applications in fields such as farmland monitoring, scientific research analysis, and pre-harvest screening, providing a powerful tool for agricultural researchers.

Aiming at the integrated operation requirements of crop–weed differentiation, disease identification and distance measurement in field conditions, the Agribot precision agricultural robot integrates the YOLO and Darknet frameworks [102]. As a single-stage convolutional neural network, it only requires one forward pass with low computational overhead. Adopting a monocular vision scheme, the system estimates the distances of weeds and crops from the camera by means of reference images and camera focal length. Its ranging accuracy basically meets the engineering requirements for targeted herbicide spraying. Nevertheless, monocular depth estimation is susceptible to light fluctuations and changes in crop height. Ranging errors gradually increase as targets move farther away. The whole system has only been validated in small-scale test scenarios, and its operational stability for large-scale field operations remains to be further examined. Built on datasets of healthy and diseased leaves, the Flask application can judge crop disease types and realize early identification and intervention of diseases. Integrated with the above perception modules and equipped with a Delta robotic arm mechanism, this autonomous robot can perform operations including fertilization, watering and weed uprooting, delivering a complete hardware solution for precision agriculture.

In rice production, conventional mechanical weeding or blanket herbicide spraying generally suffers from high costs and insufficient operation accuracy. With the YOLOv5 machine learning framework, relevant hardware and algorithms are integrated into an intelligent weeding robot for paddy fields [37]. Equipped with actuators for herbicide spraying, weed uprooting and weed incorporation, the robot adopts ideas of intelligence and automation to cut production costs and reduce ecological pollution risks. Weeds in paddy fields are located via image processing, and the YOLOv5 model is trained with corresponding datasets to realize precise weed management. As an AI-driven robot with advanced image-recognition capability, it achieves competitive recognition accuracy and running speed, and produces better weeding effects than manual labor. Prototype field tests were carried out in rice paddies at the SOA University farm in Binjhagiri, Bhubaneswar, Odisha, India. The deviation between detected weed quantity and actual weed quantity was only 2%. The weed removal rate reached 95%, and the completion rate of weed incorporation was 90%. It should be noted that existing experiments were conducted only on flat paddy fields. No adaptability tests have been performed for muddy and undulating paddy-field environments. In addition, training data sources are relatively limited. Recognition performance may degrade when rice varieties are changed or weed-community structures in fields are altered. This research provides a referable prototype for artificial-intelligence adoption in modern agriculture, promotes agriculture toward eco-friendly and economically-sustainable development, and verifies the application value of AI-based weed-management solutions in rice-planting scenarios. Relevant technologies show potential for further industrial-oriented promotion.

Deep learning-based weed-detection algorithms commonly impose heavy computational overhead, which makes it difficult to satisfy real-time running requirements for mobile robots. To address this engineering pain point, some studies combine the YOLOv8 detection model with the ByteTrack tracking algorithm to develop an autonomous agricultural robot [39]. A dataset containing 4126 images is constructed and split into a training set, a validation set and a test set at a ratio of 8:1:1. Six types of YOLO detection models are trained and compared. After comprehensive evaluation of multiple metrics, YOLOv8 is selected as the base model, achieving a precision of 93.8%, recall of 86.5%, mAP@0.5 of 92.1%, and an inference frame rate of 18 FPS. Combined with the ByteTrack multi-object-tracking algorithm, stable tracking of field weed targets can be realized. Even so, the inference speed of 18 FPS still brings certain latency pressure for high-speed moving robot platforms. When weeds are heavily occluded or weed seedlings are nearly as tall as crop plants, tracking-ID switching failures tend to occur. Given the regional limitations of the dataset, the model’s adaptability to weed species from different regions still needs further verification. Deployed on a ROS-based autonomous agricultural-robot hardware platform, the complete YOLOv8-ByteTrack model realizes real-time weed detection and physical removal, and effectively improves the efficiency of field weed-management operations.

Constrained by terrain conditions in hilly terraced farmlands of Uttarakhand, India, large-scale agricultural machinery cannot access such areas. Conventional blanket herbicide spraying incurs high costs and causes serious environmental pollution. Targeting this special scenario of hilly-farmland weed control, a portable robot platform based on YOLOv11 is developed to perform real-time weed detection and autonomous precision spraying [38]. YOLOv11 is adopted by the system to distinguish crops, weeds and backgrounds so as to support site-specific variable-rate spraying operations. Comparative performance tests between YOLOv11 and Faster R-CNN are conducted. On the independent test set, YOLOv11 achieves a precision of 0.7516, recall of 0.8846, F1-score of 0.8127, and mAP@0.5 of 0.8894. Five-fold cross-validation confirms stable performance across different data partitions. Although Faster R-CNN yields higher recall, YOLOv11 delivers better precision and F1-score with fewer false positives and higher reliability. End-to-end field prototype experiments are implemented on terraced fields covering 0.7 ha. The robot achieves a spray hit rate of 92.4%. Compared with blanket spraying, herbicide consumption is reduced by 64.8 ± 3.1%, and agent drift reaches 7.1%. It is worth noting that the field validation was only completed within a single growing season. Light variations and crop phenological shifts caused by seasonal replacement may exert potential influences on model detection performance. Meanwhile, 7.1% agent drift still poses risks of non-target phytotoxicity, and the battery life of portable devices limits long-duration continuous operations. Field tests verify the feasibility of this hilly-area autonomous precision-spraying robot. It can cut herbicide consumption, lower operating costs and mitigate environmental impacts. Further multi-season field experiments are required to fully verify model robustness.

### 6.2. Identification of Plant Diseases and Pests

Disease and pest detection plays a crucial role in modern agricultural management, as this process directly affects crop health and yield quality. Traditional pest and disease monitoring methods are often time-consuming and labor-intensive. The application of YOLO and its variants in disease and pest identification provides an efficient solution that can quickly and accurately identify crop diseases and pests, help take timely control measures, ensure crop growth, and improve resource utilization efficiency; Meanwhile, by integrating with large molds, the intelligence level of the system is further enhanced.

This section explores the application of YOLO technology in pest and disease detection robots, with a particular emphasis on the technology’s immense potential to revolutionize pest and disease management practices. We delve into how YOLO and YOLO’s variants effectively meet these challenges.

Helicoverpa armigera outbreaks in cotton fields can trigger substantial yield losses, so rapid pest and disease identification is critical for field management. Based on the Robot Operating System (ROS), an autonomous robot solution is established for cotton pest-disease monitoring [103]. It can identify bollworm infestations and track red-disease occurrences that harm cotton output. Drones conduct large-field surveys, collect multispectral imagery and compute the Normalized Difference Vegetation Index (NDVI) to generate operation routes for ground robots for targeted monitoring and resource optimization. A cotton-specific dataset is used to train the YOLOv8 model, achieving mAP of 67.1%, precision of 67.9% and recall of 61.8% for locating pests and diseases. Still, the model performs poorly on tiny early-stage lesions. Moreover, drone-ground robot collaboration relies heavily on wireless communication, which becomes unstable in low-signal farmland, and imbalanced disease samples also limit practical performance. This work organically integrates ROS, drone remote sensing, NDVI calculation and high-performance detection models to deliver a complete solution for autonomous cotton monitoring and pest control.

To realize real-time field pest-disease monitoring for tomato, a major crop in Sudan, the Agrobot agricultural robot is developed with Raspberry-Pi hardware and YOLO algorithms [104]. Equipped with a Raspberry-Pi controller and a Pi camera for live-video field scanning, the robot uses servos to realize multi-degree-of-freedom camera rotation for full-range crop inspection. Comparative tests are carried out between YOLOv4 and Tiny-YOLOv4 in tomato disease detection. Restricted by Raspberry-Pi computing power, the lightweight Tiny-YOLOv4 suffers detection-precision degradation. Besides, 2D imaging cannot detect leaf-occluded hidden diseases, and recognition accuracy drops sharply under strong backlight and other complex light conditions. Field validations prove that the robot can effectively identify major tomato diseases and common pests, laying a foundation for efficient crop management.

### 6.3. Livestock Management

Livestock management is an important component of modern agriculture, playing a crucial role in ensuring food safety, improving production efficiency, and promoting sustainable agricultural development. With the advancement of technology, traditional livestock management methods are being replaced by intelligent and automated technologies. The intelligent monitoring system integrated with YOLO variant provides an efficient and real-time solution for animal husbandry, which can accurately identify and track animals; Meanwhile, by integrating with large-scale models, the intelligence level of the system has been further enhanced.

This section explores the application of YOLO technology in livestock management, emphasizing its immense potential to revolutionize the monitoring of livestock and the prevention of diseases. We delve into how YOLO and YOLO’s variants effectively address these challenges, including real-time monitoring of animal behavior, early disease identification, and precise counting of individual livestock. By integrating into smart agricultural systems, the YOLO algorithm can enhance the efficiency and accuracy of livestock management, thereby optimizing resource allocation and disease control strategies.

Manual inspection for dead broilers in high-level caged houses is labor intensive. To solve this issue, an inspection robot with lightweight DDC-YOLO is developed for automatic dead-chicken detection [40]. Gamma transformation and Gaussian blur are adopted for data augmentation to simulate complex chicken-house conditions. Built upon YOLOv11, DDC-YOLO employs UIB lightweight backbone, WIoU loss, SimAM attention and DBFF modules, with TensorRT-based edge inference acceleration. A human-review-assisted cloud–edge update workflow is implemented for transfer-learning-driven weight iteration to adapt to diverse breeding scenarios. It achieves 96.8% precision, 85.4% recall, 90.8% F1-score and 93.1% mAP@0.5, with reduced parameters and model size compared with vanilla YOLOv11. Deployed on real farms, it supports real-time detection and reporting. Nevertheless, cloud-dependent updating fails under poor network, and recall remains low for heavily-occluded and posture-distorted dead chickens.

Improper cow-dung disposal brings disease and respiratory risks, while manual cleaning suffers from heavy workload and health threats. A YOLOv8-based robot integrating mechatronic structures is proposed for automatic cow-dung detection and collection [105]. Trained on 200 annotated field images, YOLOv8 reaches 85% detection accuracy. The forklift-screw mechanical actuator is coordinately controlled by Raspberry Pi and Arduino Nano. It can collect up to 150 kg dung per day at low cost and exhibits advantages in safety and environmental protection. Limited by dataset scale, fragmented dung on muddy ground tends to be missed, and the mechanical collector only fits flat ground.

Manual visual inspection on large beef cattle ranches easily misses early symptoms of foot-and-mouth disease and lumpy skin disease. An autonomous field rover equipped with YOLOv8 and thermal-imaging sensors is designed for early-stage cattle-health screening [106]. YOLOv8 identifies skin lesions, postures and traumas from RGB frames; thermal sensors detect fever induced by disease or heat stress as well as oestrus behavior. Field tests obtain 0.88 mAP@0.5 and 89% thermal recognition accuracy for anomaly warning. However, low night‑time temperature and rain‑snow degrade thermal measurement accuracy; lesion detection drops sharply when cattle crowd and occlude each other.

### 6.4. Yield Prediction

With the rapid development of technology, traditional yield prediction methods are gradually being replaced by agricultural robot technology. The intelligent monitoring system integrated with advanced algorithms such as YOLO provides an innovative real-time solution for modern agriculture, which can accurately predict crop yields; meanwhile, by integrating with large-scale models, the accuracy and intelligence of predictions are further improved. These systems analyze various data throughout the entire crop growth cycle, including growth rate, climate conditions, and soil quality, using big data analysis, machine learning techniques, and the multimodal processing capabilities of large models to predict final yield. Real-time analysis of these data enables farmers to timely adjust their planting strategies, such as irrigation plans, fertilization and pest control measures, so as to improve crop yield and quality, and achieve efficient deployment through edge computing and knowledge distillation in resource constrained environments.

This section delves into the application of YOLO technology in yield forecasting, highlighting the technology’s significant potential to revolutionize yield assessment and enhance the accuracy of predictions. We provide a detailed analysis of how YOLO and YOLO’s variants effectively address the challenges in yield forecasting, including real-time tracking of crop growth conditions, identifying factors affecting yield, and accurately predicting harvests. Integrating the YOLO algorithm into smart agricultural systems can enhance the accuracy and efficiency of yield forecasting, thereby optimizing planting decisions and improving the overall benefits of agricultural production. With this advanced technological application, farmers can make more informed decisions based on precise data, such as adjusting planting patterns, irrigation, and fertilization plans, as well as timely responding to various risks that may affect yield, ultimately achieving maximum yield and sustainable use of resources.

Pollination monitoring and yield prediction are critical for greenhouse production planning. A YOLOv8-based agricultural robot system is constructed for precise pollination monitoring and yield forecasting [41]. Equipped with high-definition cameras and IoT sensors, it identifies flowers, tracks pollinators and evaluates fruit development in real time. With YOLOv8, the AP for flower recognition reaches 99.4% and pollinator-tracking AP is 98.7%. Sensed data are processed on a cloud machine learning platform to achieve 99.4% yield-prediction accuracy, 22.8% higher than manual estimation. Greenhouse tomato tests show 25.6% improvement in pollination efficiency and better fruit-setting performance. This AI-driven robot reduces labor reliance and strengthens agricultural decision-making. Future work will focus on model scalability for open-field conditions and adaptive pollination strategies.

Orchard apple yield estimation is essential before growers sign commercial contracts. A flying-robot system (FRS) is developed for automatic apple counting [107]. SSD-Mobilenet and Faster R-CNN are trained on a custom red-apple dataset of 4000 images and deployed on the flying robot for orchard-scale apple detection and counting. Comparative experiments are conducted between COCO-pre-trained models and models trained on the custom dataset with learning rates from 0.015 to 0.04. Models trained on the custom dataset achieve up to 93% accuracy; the improved Faster R-CNN obtains a loss below 0.1 with competitive detection performance. Nevertheless, wind and light interference during flight bring risks of missed detection for occluded apples inside tree canopies.

Complex illumination and fruit occlusion in natural orchards create obstacles for apple-harvesting robots. Des-YOLO v4 is proposed for apple detection under such conditions [42]. To mitigate heavy computation of original YOLOv4, a lightweight Des-YOLO structure is designed to cut parameters and boost inference speed. An AP-Loss-based classification loss function is adopted to alleviate mis-detection caused by positive-negative sample imbalance, and Soft-NMS replaces conventional NMS to reduce missed detection of overlapping apples. Tested on a self-built apple dataset, Des-YOLO v4 obtains 97.13% mAP, 90% recall and 51 FPS, balancing accuracy and speed compared with YOLOv4 and Faster R-CNN. Field harvesting tests on a self-developed robot take 8.7 s per pick and achieve a 92.9% harvesting success rate. Since experiments are carried out in relatively controlled orchard environments, further verification is required for model generalization across cultivars and fruit densities.

The application of YOLO in agriculture robots has demonstrated the technology’s transformative potential in multiple key areas. From weed detection to pest and disease monitoring, livestock management, resource management, and yield estimation, the YOLO model provides real-time and efficient solutions, significantly enhancing the intelligence and sustainability of agricultural production. Despite existing challenges, continuous research efforts hold the promise of enabling the YOLO model to play an even greater role in the future of agriculture robots. Table 11 shows the applications of YOLO in different fields, along with the objectives and methods. Table 12 shows how the YOLO algorithms solves problems in agriculture.

## 7. Open Challenges in YOLO and Large Model Integration for Agricultural Automation

Despite the remarkable success of YOLO in agricultural robots, challenges persist. The regional specificity of some datasets, hardware limitations, and the need for further research to enhance model applicability are all areas worthy of attention. When YOLO is further integrated with large models, the computational complexity, modality alignment, and potential hallucinations further increase the difficulty of system deployment.

Existing practical prototypes remain limited. Farm-LightSeek [100] integrates edge-side YOLO detection with lightweight LLM reasoning, and AgriGPT [89] combines agricultural knowledge with language-model reasoning. Multimodal prototypes such as CLIP [87], LLaVA [96], and CT-CLIP [117] have also been adapted to agricultural tasks. However, most prototypes are validated in simulation or on static datasets rather than on mobile robot hardware. Three types of failure have been reported. Modal misalignment occurs when YOLO-detected disease boundaries are misinterpreted by the language model as normal variations [118]. Hallucination occurs when the language model generates agronomically incorrect recommendations [119]. Edge deployment failure occurs when compressed multimodal models still exceed the memory or power budget of Jetson-class devices [120]. These failures motivate the specific challenges discussed in the following subsections.

### 7.1. Integrate Specific Issues

In the integration process of YOLO and LLM, a key specific issue is the challenge of modal alignment, which is the inconsistency between visual detection results and text decisions [118]. For example, YOLO as a real-time object detection algorithm may accurately identify object bounding boxes in images, but when these visual outputs are passed to LLM for semantic interpretation or decision generation, differences in modal representation space may lead to decision biases or hallucinations. This inconsistency is particularly prominent in complex scenarios, such as in agricultural robot applications, where crop disease boundaries detected by YOLO may be misunderstood by LLM as normal variations, thereby affecting the reliability of automated decision-making. Research has shown that this cross modal alignment problem arises from the heterogeneity of the training data of the underlying model and the limitations of the representation learning mechanism, and needs to be alleviated through fine-grained alignment techniques to enhance the robustness and consistency of the overall system [119].

### 7.2. Resource Constraints

In the field of agricultural robots, resource constraints pose a significant challenge, especially the limited battery capacity and computing power, which makes the deployment of large-scale models extremely difficult [90]. These models require massive computing resources and memory support during the training and inference stages to process multimodal data such as visual perception and semantic decision-making. On edge devices such as agricultural robots, however, low-power processors and limited battery life can lead to high latency, overheating, or model crashes, thereby affecting real-time automation tasks such as crop monitoring or path planning [120]. A low-power edge platform such as Jetson Nano can only achieve around 10–15 FPS for YOLOv5s at 640×640, which is far below the real-time requirement of field operations, while the model itself contains about 7.5 M parameters and 13.2 B FLOPs. This limitation is particularly severe in actual field environments, where network connections are unstable and cannot rely on cloud computing, and robots need to operate autonomously for long periods of time to cover large areas of farmland.

Lightweight YOLO variants are a direct response to these resource constraints. Table 13 compares representative lightweight variants in terms of parameters, computation, inference speed, and deployment platform.

Table 13 reveals three issues. First, lightweight variants differ substantially in model size and computation, ranging from 1.9 M parameters for YOLOv5n to 11.2 M for YOLOv8s. Second, the reported FPS values are obtained under different input resolutions, batch sizes, and optimization levels, which prevents direct comparison across studies. Third, most variants are evaluated on PC or server GPUs, and only a few are validated on Jetson-class edge devices under real field conditions. These issues indicate that, although lightweight YOLO models provide a practical path toward edge deployment, a standardized benchmark for fair comparison is still lacking. This limitation needs to be alleviated through technologies such as model quantization, knowledge distillation, or federated learning to achieve sustainable deployment and reduce environmental impact.

### 7.3. Acquisition and Annotation of Datasets

In the field of agriculture, obtaining specific image datasets has always been a challenge, mainly due to the need to collect images that cover different crops, various growth stages, and diverse environmental conditions. These images are crucial for training and optimizing deep learning models like YOLO so that they can accurately recognize and classify agriculture-related objects and phenomena [121]. For instance, researchers need to gather images that include healthy and diseased plant leaves, different types of pests, and various agricultural machinery. The collection of these images must span across different seasons and weather conditions, and also take into account the diversity in geography and climate, which increases the complexity and difficulty of data collection [122].

To date, a number of datasets have been developed to support YOLO-based agricultural research. Table 14 summarizes representative examples, including their crop or animal types, sample sizes, annotation types, sensing modalities, availability, applications, and major limitations. PlantVillage, for instance, provides approximately 54K images covering 14 crops and 38 disease classes, but it was captured under laboratory conditions without field background. DeepWeeds contains 17,509 images of eight Australian weed classes, while CottonWeedDet12 provides 5648 images of 12 cotton weed classes. OpenWeedGUI offers a video dataset for green onion weeds together with an open-source annotation tool. In addition, many studies rely on self-built datasets that are rarely made public, and Roboflow Universe provides partially public agricultural datasets with inconsistent quality. These datasets differ substantially in scale, annotation style, sensing modality, and accessibility, which partly explains the limited cross-study comparability in current agricultural YOLO research.

Precision annotation of these images is also a labor-intensive and costly process [123]. Each image needs to be carefully examined by professionals and marked with regions of interest, such as the locations of pests and diseases, and the growth conditions of crops [124]. This process is not only time-consuming but also requires a high level of expertise to ensure the accuracy and consistency of the data, which is crucial for the success of subsequent model training. Moreover, due to the complexity of agricultural scenes, the development and application of automated annotation tools also face significant challenges, further increasing the workload and cost during the data preparation phase [125].

### 7.4. Model Generalization and Domain Adaptation

Agricultural vision models face severe domain shift problems in real-world deployment. A model trained on one crop, region, or season often fails to generalize to another because illumination, soil background, canopy structure, and pest morphology vary considerably across fields. Transfer learning and domain adaptation can reduce feature distribution differences between source and target domains, while self-supervised pre-training on large unlabeled field imagery can learn more general visual representations. Few-shot learning further enables rapid adaptation to rare pest categories from limited samples, and GAN-based synthetic data [28] can supplement training data for scenarios that are difficult to capture in the field. Federated learning offers an additional path by enabling model knowledge sharing across farms while protecting data privacy. Currently, these techniques remain exploratory in agricultural YOLO applications, and systematic cross-crop and cross-region validation is still lacking.

### 7.5. Real-Time Monitoring and Processing Speed

Despite the popularity of the YOLO algorithm for its efficient detection speed, in real-time agricultural monitoring, the volume of image data that need to be processed is often very large. This not only poses higher demands on the computational efficiency of the algorithm but also challenges the performance of processing devices. In resource-constrained environments, such as edge computing devices, the YOLO algorithm requires further lightweighting and optimization to reduce computational load and increase processing speed [126].

To meet the demands of real-time applications, the YOLO algorithm needs to be continuously optimized. This includes designing lighter network structures to reduce computational load and improve inference speed, as well as leveraging multi-core CPUs or GPUs for parallel computing to process multiple frames simultaneously, thereby increasing overall throughput [127]. In addition, model compression techniques are also used to significantly reduce the model size while ensuring accuracy, thereby reducing inference latency [128].

## 8. Future Directions and Opportunities

### 8.1. Technical Future Directions

The future direction of agricultural big model technology will focus on optimizing the model itself, deeply integrating multiple modalities, and improving the overall efficiency of AI. Specifically, model optimization will explore the deep integration of lightweight object detection models such as YOLO series with large-scale language/visual models to enhance modal alignment capabilities. Multimodal integration aims to build real-time frameworks such as “YOLO+Visual Language Model”, which can generate structured semantic reports while completing detection [129]. AI efficiency improvement requires a combination of technologies such as edge computing, knowledge distillation and self-monitoring learning to cope with the constraints of computing power, network and data annotation in the agricultural field [130].

### 8.2. Data and System Future Directions

The future development of agricultural big models will highly rely on collaborative innovation at the data and system levels. On the data side, the core task is to build high-quality, large-scale, and open collaborative agricultural datasets, and may promote the formation of the International Agricultural AI Data Alliance. On the system side, it is necessary to develop software frameworks that are compatible with low-power hardware to support efficient cloud–edge hybrid computing paradigms [131]. At the same time, model interpretability is crucial and requires the integration of interpretable AI technology to reveal the internal logic of decisions such as YOLO and large-scale model fusion, thereby reducing risks and establishing trust. These directions all point towards a more sustainable, reliable, and efficient agricultural AI ecosystem.

### 8.3. Application-Specific Future Directions

The future direction at the application level will focus on expanding the landing scenarios and automation depth of agricultural large-scale models, with the core goal of achieving more accurate, universal, and autonomous intelligent agriculture. Specifically, it is necessary to expand from a single task to complex scenarios such as the collaborative management of unmanned aerial vehicles and livestock health monitoring [132,133] while studying the transfer learning ability across crops and environments to improve the generalization and economy of the model [134]. More importantly, it is important to promote the deep integration of real-time perception models such as YOLO with robot actuators and large-scale language models, and build a real-time automation system that can achieve hand–eye coordination and dynamic path planning [135]. Ultimately, these technological advancements will directly serve the ultimate goal of precision agriculture: to achieve personalized, on-demand variable operations driven by architectures such as YOLO+GPT, maximizing resource utilization efficiency while minimizing environmental impact.

## 9. Conclusions

This review systematically analyzed 137 relevant studies concerning YOLO-based vision perception for agricultural robots. From the quantitative analysis of the included literature, YOLOv5 and YOLOv8 represent the most widely adopted backbones for agricultural robotic perception tasks, while research scenarios have evolved from early-stage weed and disease detection toward livestock monitoring, fruit yield estimation, and the closed-loop actuation of agricultural robots. Even so, the vast majority of existing investigations remain confined to algorithm validation on offline datasets or PC-GPU simulation environments, and only a small fraction of works have been fully deployed on real robotic hardware to achieve field closed-loop control. Several practical bottlenecks still hinder real-world field deployment. Many models suffer noticeable performance degradation when transferred across different farm sites, crop varieties, seasons and illumination conditions due to domain-shift effects. Public agricultural datasets commonly carry geographic or crop-specific bias, and high-quality open multimodal datasets reflecting complex real-field conditions remain scarce. In addition, hardware-related constraints persist, as high-performance YOLO variants cannot operate stably under real-time requirements on low-power edge devices such as Jetson-series boards. Current performance assessments predominantly rely on mAP and FPS, whereas robot-relevant practical metrics including end-to-end latency, target localization error, actuator response delay and task success rate are seldom reported in existing publications. Important unresolved research gaps also remain to be addressed. Integration pipelines connecting lightweight YOLO detectors and multimodal large vision–language models are mostly limited to conceptual validation, and robust cross-modal alignment for agricultural field tasks lacks sufficient verification under genuine farm operating conditions. There are few systematic studies exploring federated learning to handle data-privacy restrictions for multi-farm collaborative model training, and most improved YOLO variants lack supporting evidence from long-term multi-season field trials. Building on these observations, this work outlines several practical future research priorities without resorting to broad claims about agricultural modernization. Community efforts should be directed toward building open-access, multi-crop and multi-season benchmark datasets that mirror authentic complex farmland contexts to mitigate dataset bias. It is also necessary to develop lightweight YOLO optimization schemes tailored for agricultural edge hardware and place greater emphasis on robot-oriented evaluation metrics instead of over-reliance on mAP-centered offline benchmark results. Further, real-world long-duration field validation across diverse seasons and locations should be encouraged to move beyond purely laboratory-based testing. Researchers need to continue exploring robust multimodal alignment strategies for combining lightweight YOLO and vision–language large models within practical agricultural scenarios, alongside privacy-preserving training paradigms such as federated learning to support model sharing across separate farm sites.

## Figures and Tables

**Table 1:** An overview of the symbols and abbreviations.

Abbreviation	Full Name
YOLO	You Only Look Once
mAP	mean average precision
FPS	Frames Per Second
IoU	Intersection over Union
NMS	Non-Maximum Suppression
CNN	Convolutional Neural Network
FPN	Feature Pyramid Network
PAN	Path Aggregation Network
SPP	Spatial Pyramid Pooling
CSP	Cross Stage Partial
LLM	Large Language Model
ViT	Vision Transformer
UAV	Unmanned Aerial Vehicle
LiDAR	Light Detection and Ranging
V-SLAM	Visual Simultaneous Localization and Mapping
GAN	Generative Adversarial Network
RAG	Retrieval-Augmented Generation
HMI	Human–Machine Interface
RGB	Red-Green-Blue
IoT	Internet of Things
CBAM	Convolutional Block Attention Module

**Table 2:** Inclusion and exclusion criteria for literature screening.

Inclusion Criteria	Exclusion Criteria
(1) English-language journal papers or conference papers published between 2016 and 2026;	(1) Non-English literature;
(2) Using the YOLO series algorithm or its variants;	(2) Papers only reviewing YOLO algorithms without agricultural applications;
(3) Involving agriculture-related application scenarios or agricultural robot systems;	(3) Papers only involving general computer vision without agricultural scenarios;
(4) Containing quantitative performance metrics or explicit application descriptions.	(4) Papers where full text is unavailable;
	(5) Duplicate publications.

**Table 3:** Reported non-visual indicators in agricultural robot studies.

Application	End-to-End Latency	Localization Error	Control Frequency	Task Success Rate	Reference
Rice weed removal	Not reported	2% weed deviation	Not reported	95% weed removal	[37]
Hilly weed spraying	142 ± 18 ms	Not reported	Not reported	92.4% spray hit	[38]
Weed detection robot	∼60 ms	Not reported	Not reported	Not reported	[39]
Dead broiler detection	Not reported	Not reported	Not reported	92.34% field precision	[40]
Pollination and yield	Not reported	Not reported	Not reported	99.4% yield prediction	[41]
Apple harvesting	8.7 s per pick	Not reported	Not reported	92.9% harvesting	[42]

**Table 6:** Variant comparison of YOLOv5.

Model	Average Precision (@50)	Parameters	FLOPs
YOLOv5s	55.8%	7.5M	13.2B
YOLOv5m	62.4%	21.8M	39.4B
YOLOv5l	65.4%	47.8M	88.1B
YOLOv5x	66.9%	86.7M	205.7B

**Table 7:** YOLOv8 variant comparison.

Model	Size	Parameters (M)	FLOPs (G)	mAP
YOLOv8n	640	3.2	8.7	37.3%
YOLOv8s	640	11.2	28.6	44.9%
YOLOv8m	640	25.9	78.9	50.2%
YOLOv8l	640	43.7	165.2	52.9%
YOLOv8x	640	68.2	257.8	53.9%

**Table 8:** Evaluation metrics for object detection algorithms.

Evaluation Metrics	Meaning	Application Scenarios
Accuracy	Accuracy refers to the proportion of all correctly detected samples to the total sample, and the higher the value, the more reliable the overall judgment of the model.	Accuracy is applicable to agricultural scenarios where the category distribution is relatively balanced and the costs of false positives and false negatives are similar.
Precision	Precision refers to the proportion of samples predicted as positive by the model that are truly positive; the higher the value, the fewer false positives.	Precision is suitable for agricultural scenarios where missing the target is better than making errors.
Recall	Recall refers to the proportion of all true positive samples that have been successfully detected; the higher the value, the fewer missed detections. Recall is suitable for agricultural scenarios where false positives are better than missing true positives.	
Mean average precision (mAP)	mAP averages the AP of each category and measures both high recall and high precision using a 0–1 value.	The typical agricultural application scenario of mAP is object detection tasks with multiple categories, scales, and complex backgrounds.
Intersection over Union (IoU)	IoU quantifies the degree of overlap between the predicted box and the real box using a value of 0–1. The larger the value, the more accurate the positioning.	The core application scenario of IoU is agricultural detection tasks that require precise target localization.
Frames Per Second (FPS)	FPS measures the number of frames per second that a model can process, with higher values indicating better real-time performance.	The core application scenario of FPS is real-time agricultural operations that must perform “simultaneous acquisition and computation.”

**Table 9:** The solutions to key issues related to small target detection and the balance between accuracy and speed.

Key Issues	Solutions	Reference
Small object detection problem	(1) Introduce multi-scale feature fusion modules (such as FPN, PANet) between YOLO’s backbone network and detection head to enhance the feature extraction capability for small targets.	[70]
	(2) Increase the input image resolution, such as from 640 × 640 to 1280 × 1280, or use adaptive scaling strategies to avoid small targets losing details during downsampling.	[53]
	(3) Add attention modules such as Convolutional Block Attention Module (CBAM) and Squeeze-and-Excitation Block (SE Block) to YOLO’s backbone network to enhance attention to key features of small targets.	[71]
The balance between accuracy and speed	(1) Replace standard CNN with more efficient backbone networks such as ShuffleNet, MobileNet, and EfficientNet to reduce computational complexity while maintaining high detection accuracy.	[72]
	(2) Reduce model size and computational complexity through 8-bit/4-bit quantization or channel pruning, and improve inference speed.	[73]
	(3) Using dynamic resolution input or Early Exit mechanism, adjust the computational complexity according to the input complexity, such as using low resolution for simple backgrounds and high resolution for complex scenes.	[74,75]

**Table 10:** Various YOLO-based models.

Model	YOLO Versions	Methods	Advantage
Tinier-YOLO [76]	YOLOv3	Introduce SqueezeNet’s Fire module, DenseNet style dense connections, and Passthrough layer in Tiny-YOLO-V3, and remove BatchNorm from the Fire module.	The parameter size has been reduced to 8.9 MB, Jetson TX1 has been tested at 25 FPS, and VOC mAP is 65.7%, balancing small size, high speed, and high precision.
YOLO + MSCIM [77]	YOLOv4	Introducing a multi-scale contextual information fusion module in the YOLOv4 framework to enhance the feature representation of small targets.	Significantly improve the accuracy of small target detection while maintaining real-time performance.
HIC-YOLOv5 [60]	YOLOv5	Add 160 × 160 high-resolution SODH on YOLOv5, insert CFFI between backbone necks, and embed lightweight CBAM attention at the end of the backbone.	On VisDrone2019, mAP increased by 9.38%, mAP increased by 6.42%, and the parameter count was only 8.39 M. The real-time performance was better than models of the same scale.
MSC-YOLO [78]	YOLOv7	Add a 160 × 160 “Tiny“ prediction header on the basis of YOLOv7; Replace downsampling with CSPDC to reduce detail loss; Add SCPMA fusion to multi-scale spatial channel context.	On VisDrone2023 and UAVDT, mAP increased by 3% compared to YOLOv7, small target mAPs increased by 2.8%, and only increased parameters by 8.9%, maintaining real-time performance.
YOLO + DIOU [79]	YOLOv5	Add a small object detection layer of 160 × 160 in YOLOv5s and replace the traditional NMS suppression box with DIOU-NMS.	On VisDrone2019, mAP increased by 10.9%, recall increased by 9.8%, and single-stage real-time operation outperformed Faster R-CNN.

**Table 11:** Application areas of YOLO in agriculture robots.

Domain	Goal	Method	Reference
Weed detection	Realize integrated crop–weed discrimination, disease identification and distance measurement for field robots.	(1) Adopt Agribot robot integrating YOLO and Darknet. Monocular vision is used for distance estimation; Flask application is built for disease diagnosis. Delta robotic arm performs fertilization and weed removal. Only small-scale field tests are completed.	[102]
	Build intelligent weeding robot for paddy fields to reduce herbicide consumption and production cost.	(2) Integrate YOLOv5 with spraying, uprooting and incorporation actuators. Field prototype test is carried out in rice paddy. Weed quantity deviation is only 2%.	[37]
	Develop autonomous agricultural robot for real-time weed detection and physical removal.	(3) Combine YOLOv8 and ByteTrack for weed detection and tracking. Model is trained on 4126 images and deployed on ROS-based mobile robot platform.	[39]
	Develop portable robot for hilly terraces to achieve site-specific precision herbicide spraying.	Adopt YOLOv11 to distinguish crops, weeds and background. End-to-end field test is conducted on 0.7 ha terraced farmland. Herbicide usage is greatly reduced.	[38]
Identification of plant diseases and pests	Build autonomous robot system for cotton bollworm and red disease monitoring in large fields.	(1) Deploy ROS-based ground robot combined with UAV multispectral imaging. NDVI is calculated for path planning. YOLOv8 is trained on cotton-specific dataset.	[103]
	Realize real-time pest-disease monitoring for field tomato in Sudan.	(2) Construct Agrobot based on Raspberry Pi. Compare YOLOv4 and Tiny-YOLOv4. Pan-tilt Pi-camera is used for full-range crop scanning.	[104]
Livestock Management	Develop inspection robot for automatic dead-broiler detection in caged poultry houses.	(1) Propose DDC-YOLO based on YOLOv11 with UIB backbone, WIoU, SimAM and DBFF module. TensorRT edge acceleration and human-review-aided cloud-edge update workflow are adopted.	[40]
	Build robot for automatic cow dung detection and collection on livestock farm.	(2) YOLOv8 is trained on 200 field images. Forklift-screw mechanical structure is controlled by Raspberry Pi and Arduino Nano for dung collection.	[105]
	Realize early-stage health anomaly warning for beef cattle in open pastures.	(3) Design field rover integrating YOLOv8 and infrared thermal imager. Detect skin lesions and body-surface temperature changes for disease and oestrus judgement.	[106]
Yield prediction	Implement greenhouse pollination monitoring and accurate crop yield prediction.	(1) Agricultural robot equipped with HD cameras and IoT sensors runs YOLOv8. Cloud-side machine learning platform analyzes perception data for yield forecasting. Greenhouse tomato experiment is performed.	[41]
	Achieve automatic apple counting for orchard yield estimation before commercial contracts.	(2) Flying-robot system (FRS) deploys SSD-Mobilenet and Faster R-CNN, which are trained on custom dataset containing 4000 red-apple images for orchard on-site test.	[107]
	Propose apple detection algorithm for harvesting robot under complex orchard occlusion condition.	(3) Des-YOLO v4 lightweight network is proposed. AP-Loss and Soft-NMS replace original NMS. Tests are finished on self-built apple dataset and physical picking robot platform.	[42]

**Table 12:** YOLO algorithms to solve problems in agriculture.

Problem	Solution	Reference
There are numerous types and significant morphological differences of weeds in farmland, and traditional methods are difficult to identify with high accuracy, which affects the effectiveness of precise weed control.	(1) In YOLOv8, GFPN multi-scale feature fusion, multi EAM attention mechanism, and Slide Loss are introduced to improve the accuracy of maize seedling detection. Then, OpenCV is used to extract weeds in reverse, achieving real-time weed recognition with an accuracy of 92.9%.	[108]
There are various types of crop pests and diseases, complex symptoms, low efficiency of manual identification, and easy misjudgment.	(1)Propose a YOLO-JD model that integrates hourglass feature extraction and pyramid pooling, and train the model on a self built large-scale image dataset to achieve high-precision real-time disease and pest detection.	[109]
	(2) Propose lightweight AgriPest YOLO, embed CLA and GSPPF in YOLOv5s, and use Soft NMS post-processing to achieve real-time and accurate detection with 71.3% mAP and 8.8ms inference on 25k 24 class pest datasets.	[110]
	(3) Build a 10,000 level image dataset and achieve real-time pest recognition and localization with 92.86% accuracy using YOLOv4, which integrates DIoU NMS, secondary fine-tuning, and data augmentation.	[111]
Pigs in the pigsty often come into close contact, and traditional methods are difficult to accurately distinguish individuals in real time, which affects all-weather health monitoring.	(1) First, use infrared and depth information to detect the “contact pig herd”, then use YOLO to quickly locate the contact boundary, and finally perform image processing segmentation to achieve real-time and high-precision single pig separation.	[112]
The frequent crawling and crossing behavior of cows at night is caused by severe scale changes due to insufficient lighting and differences in shooting distance. Traditional methods are difficult to detect in real-time and accurately, which affects estrus recognition and farm economic benefits.	(1) Propose the IATEFF-YOLO model, enhance nighttime low light images using an “adaptive lighting transformer”, and combine the enhanced image data with an “efficient feature fusion detector” to introduce SPPCSPC and Selective Kernel Attention mechanisms in the YOLO X framework, achieving real-time and accurate crawling behavior detection with 99.3% mAP and 102 f/s.	[113]
In complex cowshed environments, traditional multi-target tracking algorithms suffer from low detection accuracy and frequent identity switching when dealing with issues such as occlusion between cows, facility occlusion, and lighting changes, making this situation difficult to accurately identify and track the behavior of multiple cows.	(1) A multi-objective behavior recognition and tracking model based on improved YOLOv8 and BoTSORT algorithms was proposed. By introducing improvement measures such as dynamic convolution (DyConv), C2f iRMB structure, Adown downsampling module, and dynamic detection head (DyHead), the detection accuracy and tracking stability were improved, and a virtual trajectory update mechanism was adopted to reduce the accumulation of prediction errors, thus achieving stable tracking and long-term behavior analysis of cattle.	[114]
Counting soybean pods is difficult due to the inefficiency of traditional methods and the insufficient accuracy of existing technologies.	(1) A YOLO POD model based on an improved YOLO X framework was proposed. By adding a module for predicting the number of pods, modifying the loss function, and introducing a CBAM, a multi task model was constructed to achieve accurate identification and counting of soybean pods without reducing inference speed.	[115]
The Central Support Axis Irrigation System (CPIS) has technical issues in the system’s automatic control system, resulting in damage to the main pipelines and towers, causing significant losses to farmers and crops, and the repair process is time-consuming.	(1) Using the YOLOv7 algorithm to detect defects in CPIS machines, real-time monitoring and control of irrigation system defects can be achieved by identifying whether they are in a safe or dangerous state.	[116]

**Table 13:** Horizontal comparison of lightweight YOLO variants for edge deployment.

Model	Params	FLOPs	Reported FPS	Deployment Platform	Reference
YOLOv5n	1.9 M	4.5 G	10–15	Jetson Nano	[9]
YOLOv5s	7.5 M	13.2 G	30–40	Jetson Xavier NX	[9]
YOLOv8n	3.2 M	8.7 G	15–25	Jetson Nano/RPi + Coral	[54]
YOLOv8s	11.2 M	28.6 G	40–60	Jetson Xavier NX	[54]
Tinier-YOLO	8.9 MB	Not reported	25	Jetson TX1	[76]
DDC-YOLO	Reduced by 1.437 M	Not reported	43 (PyTorch 1.13)/ 31.7 (TensorRT 8.5)	Jetson Orin NX	[40]

Note: Reported FPS values are obtained under different input resolutions, batch sizes, and optimization levels, and are therefore indicative rather than directly comparable.

**Table 14:** Representative agricultural datasets used in YOLO research.

Dataset	Classes	Size	Annotation Type	Sensing Modality	Application	Limitations
PlantVillage	14 crops, 38 disease classes	∼54K images	Class labels	RGB	Disease detection	Lab environment, no field background
DeepWeeds	8 Australian weed classes	17,509 images	Bounding boxes	RGB	Weed detection	Region-specific weeds
CottonWeedDet12	12 cotton weed classes	5648 images	Bounding boxes	RGB	Weed detection	Cotton field only
OpenWeedGUI	Green onion weeds	Video dataset	Bounding boxes	RGB	Weed detection	Single crop type
Self-built datasets	Various	Hundreds to thousands	Dataset-specific	RGB/ multispectral/thermal	Dataset-specific	Poor reproducibility
Roboflow Universe	Various agricultural	Tens of thousands	Bounding boxes/masks	RGB	Various	Inconsistent quality

Note: Annotation types include bounding boxes, class labels, and segmentation masks depending on the dataset. Sensing modalities include RGB, multispectral, and thermal imagery.

## Data Availability

Data sharing is not applicable to this article as no new data were created or analyzed in this study.
